# Supplementary material for: Efficient protein structure archiving using ProteStAr
Source: Bioinformatics. 2024 Jul 10;40(7):btae428. doi: 10.1093/bioinformatics/btae428 (PMC11239224; doi:10.1093/bioinformatics/btae428)
Supplement: btae428_Supplementary_Data [file btae428_supplementary_data.zip › ProteStAr_supp.pdf]

Supplementary material for
Efficient protein structure archiving using ProteStAr

Sebastian Deorowicz   Adam Gudyś

June 11, 2024

Contents

1 Datasets **2**
- 1.1 Model organisms proteomes from APSD . . . 2
- 1.2 Human proteome (large) . . . 2
- 1.3 ESM Atlas — full datasets . . . 2
- 1.4 ESM Atlas — subset . . . 2

2 Examined programs **3**
- 2.1 gzip v. 1.12 . . . 3
- 2.2 BinaryCIF, CIFTools v. 5.0.0 . . . 3
- 2.3 Foldcomp v. 0.0.5 . . . 5
- 2.4 PDC commit c49393d (Mar 7, 2023) . . . 5
- 2.5 ProteStAr v. 1.1.0 . . . 6
- 2.6 tar v. 1.34 . . . 6

3 Environment **7**

4 Additional results **8**

5 Largest coordinate reconstruction errors **10**
- 5.1 Foldcomp . . . 10
- 5.2 PDC . . . 15
- 5.3 ProteStAr 10/10 . . . 20
- 5.4 ProteStAr 10/100 . . . 25
- 5.5 ProteStAr 80/140 . . . 30
- 5.6 ProteStAr 200/300 . . . 35

1 Datasets

We used datasets from AlphaFold Protein Structures Database v.4 and ESM Atlas v0 and v2023_02.

1.1 Model organisms proteomes from APSD

Data downloaded from `https://alphafold.ebi.ac.uk/download`.

- Budding yeast
  `https://ftp.ebi.ac.uk/pub/databases/alphafold/latest/UP000002311_559292_YEAST_v4.tar`
- *E.coli*
  `https://ftp.ebi.ac.uk/pub/databases/alphafold/latest/UP000000625_83333_ECOLI_v4.tar`
- Human
  `https://ftp.ebi.ac.uk/pub/databases/alphafold/latest/UP000005640_9606_HUMAN_v4.tar`
- Maize
  `https://ftp.ebi.ac.uk/pub/databases/alphafold/latest/UP000007305_4577_MAIZE_v4.tar`
- Mouse
  `https://ftp.ebi.ac.uk/pub/databases/alphafold/latest/UP000000589_10090_MOUSE_v4.tar`
- Swiss-Prot CIF
  `https://ftp.ebi.ac.uk/pub/databases/alphafold/latest/swissprot_cif_v4.tar`
- Swiss-Prot PDB
  `https://ftp.ebi.ac.uk/pub/databases/alphafold/latest/swissprot_pdb_v4.tar`

1.2 Human proteome (large)

The variant of human proteome with PAE and confidence files was downloaded from Google Cloud Public Datasets as described at: `https://alphafold.ebi.ac.uk/download`.

1.3 ESM Atlas — full datasets

The full datasets are downloaded from `https://github.com/facebookresearch/esm/tree/main/scripts/atlas`. We followed the links from `https://github.com/facebookresearch/esm/blob/main/scripts/atlas/v0/full/tarballs.txt` (v0 version) and `https://github.com/facebookresearch/esm/blob/main/scripts/atlas/v2023_02/full/tarballs.txt` (v2023_02 version).

1.4 ESM Atlas — subset

For evaluation purposes we picked approx. 59k PDB files from ESM Atlas. Details how to download this dataset are given at: `https://github.com/refresh-bio/protestar`.

2 Examined programs

The following programs were used in the experimental part. Running parameters are also given.

2.1 gzip v. 1.12

- Compression of a directory

```
# <dataset_name> <in_dir> <out_dir> <tmp_dir>
for i in $2/*; do mv "$i" $4; done
gzip -k -9 $4/*.cif
tar -cf $3/$1.tar $4/*.gz
```

- Decompression of an archive

```
# <dataset_name> <in_dir> <out_dir> <tmp_dir>
tar -xf $3/$1.tar
gzip -d $4/*.gz
```

2.2 BinaryCIF, CIFTools v. 5.0.0

- Compression — we implemented a short script for bulk compression of many CIF files:

```
package org.rcsb.cif;

import org.rcsb.cif.model.CifFile;
import org.rcsb.cif.model.FloatColumn;
import org.rcsb.cif.schema.StandardSchemata;
import org.rcsb.cif.schema.mm.AtomSite;
import org.rcsb.cif.schema.mm.MmCifBlock;
import org.rcsb.cif.schema.mm.MmCifFile;

import java.io.IOException;
import java.net.URL;
import java.util.Optional;
import java.util.OptionalDouble;
import java.util.OptionalInt;
import java.io.File;
import java.nio.file.Path;
import java.nio.file.Paths;

public class CompressBatch {
    public static void main(String[] args) throws Exception {
        // Get args
        // args[0] = input directory that contains cif files
        // args[1] = output directory that will contain bcif files
        Path input = Paths.get(args[0]);
        Path output = Paths.get(args[1]);
        // Get all files in input directory
        File[] files = input.toFile().listFiles();
        // Loop through all files

        for (File file : files) {
            // Get file name
            String fileName = file.getName();
            // Get file extension
            String fileExtension = fileName.substring(fileName.lastIndexOf(".") + 1, fileName.length());
            String outputFileName = fileName.substring(0, fileName.lastIndexOf(".")) + ".bcif";
            // Check if file is a cif file
            if (fileExtension.equals("cif")) {
                // Get input file path
                Path inputFilePath = Paths.get(input.toString(), fileName);
                // Get output file path
```

```
                Path outputFilePath = Paths.get(output.toString(), outputFileName);
                // Measure running time
                long endTime;
                long startTime = System.nanoTime();
                // Handle exceptions
                try {
                    // Read cif file
                    CifFile cifFile = CifIO.readFromPath(inputFilePath);
                    // Write bcif file
                    CifIO.writeBinary(cifFile, outputFilePath);
                    endTime = System.nanoTime();
                    System.out.println(fileName + "\t" + (endTime - startTime) / 1000000000.0);
                } catch (Exception e) {
                    System.out.println(fileName+ "\t" + "NA");
                }
            }
        }
    }
}
```

- Decompression — we implemented the following script

```
package org.rcsb.cif;

import org.rcsb.cif.model.CifFile;
import org.rcsb.cif.model.FloatColumn;
import org.rcsb.cif.schema.StandardSchemata;
import org.rcsb.cif.schema.mm.AtomSite;
import org.rcsb.cif.schema.mm.MmCifBlock;
import org.rcsb.cif.schema.mm.MmCifFile;

import java.io.IOException;
import java.net.URL;
import java.util.Optional;
import java.util.OptionalDouble;
import java.util.OptionalInt;
import java.io.File;
import java.nio.file.Path;
import java.nio.file.Paths;

public class DecompressBatch {
    public static void main(String[] args) throws Exception {
        // Get args
        // args[0] = input directory that contains bcif files
        // args[1] = output directory that will contain cif files
        Path input = Paths.get(args[0]);
        Path output = Paths.get(args[1]);
        // Get all files in input directory
        File[] files = input.toFile().listFiles();
        // Loop through all files
        for (File file : files) {
            // Get file name
            String fileName = file.getName();
            // Get file extension
            String fileExtension = fileName.substring(fileName.lastIndexOf(".") + 1, fileName.length());
            String outputFileName = fileName.substring(0, fileName.lastIndexOf(".")) + ".cif";
            // Check if file is a cif file
            if (fileExtension.equals("bcif")) {
                // Get input file path
                Path inputFilePath = Paths.get(input.toString(), fileName);
                // Get output file path
                Path outputFilePath = Paths.get(output.toString(), outputFileName);
                // Measure running time
                long endTime;
                long startTime = System.nanoTime();
                // Handle exceptions
                try {
                    // Read bcif file
```

```
                    CifFile cifFile = CifIO.readFromPath(inputFilePath);
                    // Write cif file
                    CifIO.writeText(cifFile, outputFilePath);
                    endTime = System.nanoTime();
                    System.out.println(fileName + "\t" + (endTime - startTime) / 1000000000.0);
                } catch (Exception e) {
                    System.out.println(fileName+ "\t" + "NA");
                }
            }
        }
    }
}
```

2.3 Foldcomp v. 0.0.5

- Compression of a directory

```
# <dataset_name> <in_dir> <out_dir> <tmp_dir>
./utils/foldcomp compress -t 16 $2 --db -y $3/$1.fcz
```

- Decompression of an archive

```
# <dataset_name> <in_dir> <out_dir> <tmp_dir>
./utils/foldcomp decompress -t 16 $3/$1.fcz $4
```

2.4 PDC commit c49393d (Mar 7, 2023)

- Compression of a directory—lossless

```
# <dataset_name> <in_dir> <out_dir> <tmp_dir>
for f in $2/*.cif; do
./utils/pdc $f $4/$(basename $f).pdc -l=0
done
tar -cf $3/$1.pdc_lossless_tar $4/*.pdc
```

- Compression of a directory—lossy

```
# <dataset_name> <in_dir> <out_dir> <tmp_dir>
for f in $2/*.cif; do
./utils/pdc $f $4/$(basename $f).pdc -l=2
done
tar -cf $3/$1.pdc_lossy_tar $4/*.pdc
```

- Decompression of an archive—lossless

```
# <dataset_name> <in_dir> <out_dir> <tmp_dir>
tar -xf $3/$1.pdc_lossless_tar
for f in $4/*.pdc; do
./utils/pdd $f $4/$(basename $f).cif -f=2
done
```

- Decompression of an archive—lossy

```
# <dataset_name> <in_dir> <out_dir> <tmp_dir>
tar -xf $3/$1.pdc_lossy_tar
for f in $4/*.pdc; do
./utils/pdd $f $4/$(basename $f).cif -f=2
done
```

2.5 ProteStAr v. 1.1.0

- Compression of a directory—lossless

```
# <dataset_name> <in_dir> <out_dir> <tmp_dir>
./utils/psarch add --type pdb -t 16 --indir $2 --out $3/$1.psarch_pdb_lossless -v 0
```

- Compression of an archive—minimal

```
# <dataset_name> <in_dir> <out_dir> <tmp_dir>
./utils/psarch add --type pdb -t 16 --indir $2 --out $3/$1.psarch_minimal -v 0 --minimal
```

- Compression of an archive—10/10

```
# <dataset_name> <in_dir> <out_dir> <tmp_dir>
./utils/psarch add --type pdb -t 16 --indir $2 --out $3/$1.psarch_minimal_10_10 -v 0
    --minimal --lossy --max-error-bb 10 --max-error-sc 10
```

- Compression of an archive—10/100

```
# <dataset_name> <in_dir> <out_dir> <tmp_dir>
./utils/psarch add --type pdb -t 16 --indir $2 --out $3/$1.psarch_minimal_10_100 -v 0
    --minimal --lossy --max-error-bb 10 --max-error-sc 100
```

- Compression of an archive—80/140

```
# <dataset_name> <in_dir> <out_dir> <tmp_dir>
./utils/psarch add --type pdb -t 16 --indir $2 --out $3/$1.psarch_minimal_80_140 -v 0
    --minimal --lossy --max-error-bb 80 --max-error-sc 140
```

- Compression of an archive—200/300

```
# <dataset_name> <in_dir> <out_dir> <tmp_dir>
./utils/psarch add --type pdb -t 16 --indir $2 --out $3/$1.psarch_minimal_200_300 -v 0
    --minimal --lossy --max-error-bb 200 --max-error-sc 300
```

- Decompression of an archive

```
# <dataset_name> <in_dir> <out_dir> <tmp_dir>
./utils/psarch get --type ALL --all -t 16 --in $3/$1.psarch_pdb_lossless --outdir $4/ -v 0
```

2.6 tar v. 1.34

3 Environment

The machine used in the tests was of the following configuration:

- AMD 3995WX Pro CPU clocked at 2.7 GHz CPU, 64 cores,
- 512 GiB RAM,
- NVME Seagate FireCuda 530 4 TB—used in majority of tests,
- RAID5 composed of four Seagate Exos 16 TB HDDs—used in compression of full ESM Atlas,
- openSUSE Tumbleweed operating system.

Our tool was compiled using gcc 11.3.0.

4 Additional results

Additional results are given in the Supplementary Worksheet.

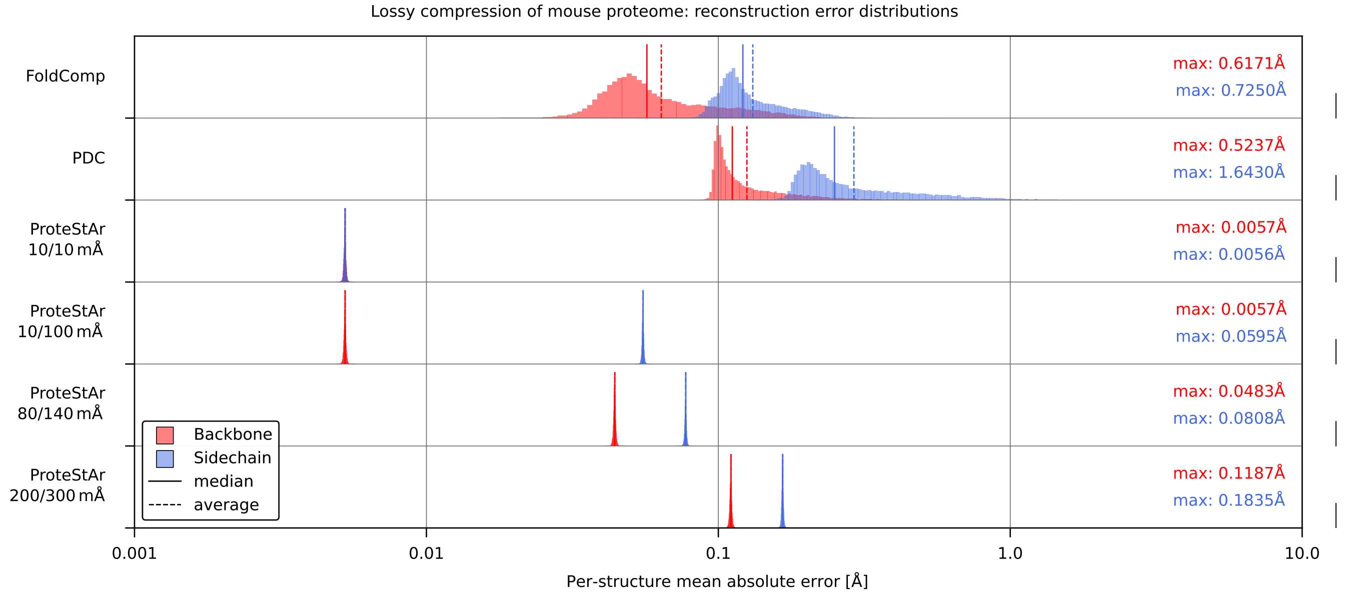

Supplementary Figure 1: Histograms of per-structure mean absolute errors (MAE) for backbone (red) and side chain (blue) atoms. Median, average, and maximum values of MAE are also presented.

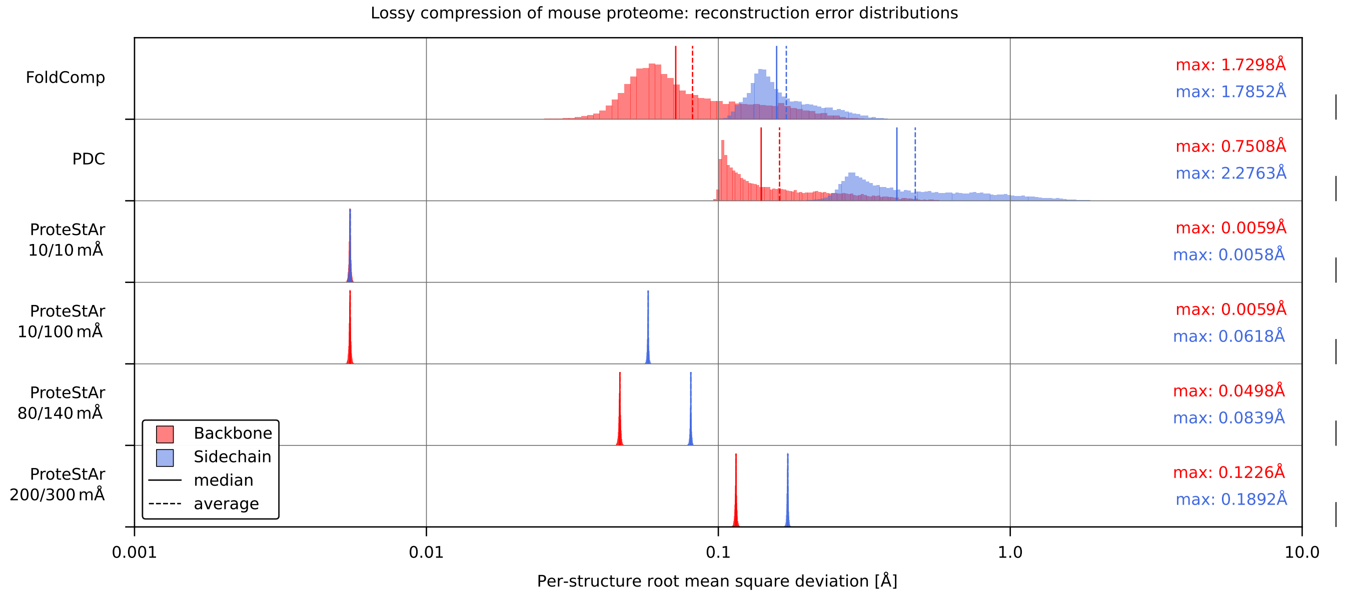

Supplementary Figure 2: Histograms of per-structure root mean square deviations (RMSD) for backbone (red) and side chain (blue) atoms. Median, average, and maximum values of RMSD are also presented.

5 Largest coordinate reconstruction errors

In this section, we list fragments of original and reconstructed PDB files from mouse proteome containing atoms with the largest reconstruction error (five for each of the investigated package configurations). The maximal errors for each structure are marked with `***`.

5.1 Foldcomp

```
*************** AF-Q9D9U9-F1-model_v4.pdb max_error: 10.7792
***** original
ATOM   2754  N   ARG A 334      34.231  34.378 -24.942  1.00 40.83           N
ATOM   2755  CA  ARG A 334      32.528  34.915 -23.929  1.00 40.83           C
ATOM   2756  C   ARG A 334      30.699  34.697 -23.103  1.00 40.83           C
ATOM   2757  CB  ARG A 334      32.393  36.441 -23.833  1.00 40.83           C
ATOM   2758  O   ARG A 334      30.706  34.269 -21.940  1.00 40.83           O
ATOM   2759  CG  ARG A 334      31.998  37.075 -22.471  1.00 40.83           C
ATOM   2760  CD  ARG A 334      32.614  36.591 -21.143  1.00 40.83           C
ATOM   2761  NE  ARG A 334      33.862  35.816 -21.251  1.00 40.83           N
ATOM   2762  NH1 ARG A 334      35.206  37.272 -22.442  1.00 40.83           N
ATOM   2763  NH2 ARG A 334      35.866  35.192 -22.057  1.00 40.83           N
ATOM   2764  CZ  ARG A 334      34.968  36.110 -21.905  1.00 40.83           C
ATOM   2765  N   LEU A 335      29.225  35.186 -23.215  1.00 56.80           N
ATOM   2766  CA  LEU A 335      27.840  35.552 -22.181  1.00 56.80           C
ATOM   2767  C   LEU A 335      26.314  34.869 -21.744  1.00 56.80           C
ATOM   2768  CB  LEU A 335      27.268  37.059 -22.376  1.00 56.80           C
ATOM   2769  O   LEU A 335      25.861  35.138 -20.623  1.00 56.80           O
ATOM   2770  CG  LEU A 335      26.966  38.158 -21.250  1.00 56.80           C
ATOM   2771  CD1 LEU A 335      26.362  39.425 -21.892  1.00 56.80           C
ATOM   2772  CD2 LEU A 335      26.057  37.944 -20.023  1.00 56.80           C
ATOM   2773  N   ALA A 336      25.381  34.131 -22.446  1.00 47.52           N
ATOM   2774  CA  ALA A 336      23.902  33.930 -22.035  1.00 47.52           C
ATOM   2775  C   ALA A 336      23.497  32.903 -20.914  1.00 47.52           C
ATOM   2776  CB  ALA A 336      23.057  33.603 -23.283  1.00 47.52           C
ATOM   2777  O   ALA A 336      22.683  33.197 -20.028  1.00 47.52           O
***** foldcomp
ATOM   2754  N   ARG A 334      43.035  33.468 -27.438  1.00 40.72           N  <-- err: 9.1961
ATOM   2755  CA  ARG A 334      41.570  33.883 -26.613  1.00 40.72           C  <-- err: 9.4882
ATOM   2756  C   ARG A 334      39.924  33.755 -25.881  1.00 40.72           C  <-- err: 9.6801
ATOM   2757  O   ARG A 334      39.800  33.353 -24.726  1.00 40.72           O  <-- err: 9.5552
ATOM   2758  CB  ARG A 334      41.970  35.350 -26.784  1.00 40.72           C  <-- err: 10.0806
ATOM   2759  CG  ARG A 334      41.783  36.179 -25.511  1.00 40.72           C  <-- err: 10.2855
ATOM   2760  CD  ARG A 334      42.240  35.400 -24.288  1.00 40.72           C  <-- err: 10.1965
ATOM   2761  NE  ARG A 334      43.099  34.276 -24.648  1.00 40.72           N  <-- err: 9.9616
ATOM   2762  CZ  ARG A 334      44.152  34.346 -25.442  1.00 40.72           C  <-- err: 9.9984
ATOM   2763  NH1 ARG A 334      44.604  35.512 -25.831  1.00 40.72           N  <-- err: 10.1442
ATOM   2764  NH2 ARG A 334      44.721  33.249 -25.875  1.00 40.72           N  <-- err: 9.8368
ATOM   2765  N   LEU A 335      38.396  34.208 -25.872  1.00 56.95           N  <-- err: 9.5981
ATOM   2766  CA  LEU A 335      36.997  34.544 -24.919  1.00 56.95           C  <-- err: 9.6106
ATOM   2767  C   LEU A 335      35.377  33.976 -24.430  1.00 56.95           C  <-- err: 9.4947
ATOM   2768  O   LEU A 335      34.926  34.268 -23.318  1.00 56.95           O  <-- err: 9.4971
ATOM   2769  CB  LEU A 335      36.962  36.038 -25.247  1.00 56.95           C  <-- err: 10.1616
ATOM   2770  CG  LEU A 335      36.864  36.987 -24.052  1.00 56.95           C  <-- err: 10.3534
ATOM   2771  CD1 LEU A 335      36.768  38.430 -24.522  1.00 56.95           C  <-- err: 10.7792 ***
ATOM   2772  CD2 LEU A 335      35.650  36.650 -23.201  1.00 56.95           C  <-- err: 10.1882
ATOM   2773  N   ALA A 336      34.191  33.295 -25.009  1.00 47.57           N  <-- err: 9.2132
ATOM   2774  CA  ALA A 336      32.532  33.143 -24.518  1.00 47.57           C  <-- err: 9.0145
ATOM   2775  C   ALA A 336      31.876  32.174 -23.339  1.00 47.57           C  <-- err: 8.7533
ATOM   2776  O   ALA A 336      31.050  32.629 -22.550  1.00 47.57           O  <-- err: 8.7573
ATOM   2777  CB  ALA A 336      31.818  32.940 -25.845  1.00 47.57           C  <-- err: 9.152
```

```
*************** AF-O70445-F1-model_v4.pdb max_error: 6.893
***** original
ATOM   1693  N   SER A 220     -16.795 -10.911  -5.968  1.00 33.04           N
ATOM   1694  CA  SER A 220     -18.014 -10.073  -5.950  1.00 33.04           C
ATOM   1695  C   SER A 220     -18.445  -9.462  -4.601  1.00 33.04           C
ATOM   1696  CB  SER A 220     -19.192 -10.588  -6.786  1.00 33.04           C
ATOM   1697  O   SER A 220     -19.074 -10.168  -3.827  1.00 33.04           O
ATOM   1698  OG  SER A 220     -19.939 -11.547  -6.087  1.00 33.04           O
ATOM   1699  N   ARG A 221     -18.178  -8.203  -4.175  1.00 37.70           N
ATOM   1700  CA  ARG A 221     -17.672  -6.899  -4.751  1.00 37.70           C
ATOM   1701  C   ARG A 221     -18.987  -5.819  -4.701  1.00 37.70           C
ATOM   1702  CB  ARG A 221     -16.419  -7.334  -5.761  1.00 37.70           C
ATOM   1703  O   ARG A 221     -19.898  -6.326  -4.082  1.00 37.70           O
ATOM   1704  CG  ARG A 221     -16.042  -7.780  -7.290  1.00 37.70           C
ATOM   1705  CD  ARG A 221     -16.924  -8.024  -8.596  1.00 37.70           C
ATOM   1706  NE  ARG A 221     -16.359  -8.434  -9.893  1.00 37.70           N
ATOM   1707  NH1 ARG A 221     -18.009  -9.984 -10.447  1.00 37.70           N
ATOM   1708  NH2 ARG A 221     -16.494  -9.442 -11.935  1.00 37.70           N
ATOM   1709  CZ  ARG A 221     -16.957  -9.268 -10.742  1.00 37.70           C
ATOM   1710  N   PHE A 222     -19.425  -4.498  -5.035  1.00 43.49           N
ATOM   1711  CA  PHE A 222     -19.195  -3.016  -5.575  1.00 43.49           C
ATOM   1712  C   PHE A 222     -20.040  -1.980  -4.762  1.00 43.49           C
ATOM   1713  CB  PHE A 222     -19.479  -2.618  -7.085  1.00 43.49           C
ATOM   1714  O   PHE A 222     -20.766  -2.358  -3.864  1.00 43.49           O
ATOM   1715  CG  PHE A 222     -18.424  -1.858  -7.913  1.00 43.49           C
ATOM   1716  CD1 PHE A 222     -18.301  -0.463  -7.894  1.00 43.49           C
ATOM   1717  CD2 PHE A 222     -17.653  -2.523  -8.876  1.00 43.49           C
ATOM   1718  CE1 PHE A 222     -17.407   0.216  -8.740  1.00 43.49           C
ATOM   1719  CE2 PHE A 222     -16.649  -1.888  -9.597  1.00 43.49           C
ATOM   1720  CZ  PHE A 222     -16.532  -0.508  -9.558  1.00 43.49           C
***** foldcomp
ATOM   1693  N   SER A 220     -16.773 -10.874  -5.939  1.00 33.15           N  <-- err: 0.0519
ATOM   1694  CA  SER A 220     -17.978 -10.040  -5.914  1.00 33.15           C  <-- err: 0.0607
ATOM   1695  C   SER A 220     -18.404  -9.431  -4.574  1.00 33.15           C  <-- err: 0.0581
ATOM   1696  O   SER A 220     -19.055 -10.093  -3.768  1.00 33.15           O  <-- err: 0.0973
ATOM   1697  CB  SER A 220     -19.204 -10.525  -6.690  1.00 33.15           C  <-- err: 0.1155
ATOM   1698  OG  SER A 220     -19.948 -11.465  -5.933  1.00 33.15           O  <-- err: 0.1747
ATOM   1699  N   ARG A 221     -18.146  -8.185  -4.150  1.00 37.67           N  <-- err: 0.0444
ATOM   1700  CA  ARG A 221     -17.667  -6.911  -4.700  1.00 37.67           C  <-- err: 0.0526
ATOM   1701  C   ARG A 221     -18.851  -5.934  -4.647  1.00 37.67           C  <-- err: 0.1861
ATOM   1702  O   ARG A 221     -19.887  -6.261  -4.069  1.00 37.67           O  <-- err: 0.0672
ATOM   1703  CB  ARG A 221     -16.906  -6.763  -6.019  1.00 37.67           C  <-- err: 0.7936
ATOM   1704  CG  ARG A 221     -17.825  -6.584  -7.229  1.00 37.67           C  <-- err: 2.1478
ATOM   1705  CD  ARG A 221     -19.285  -6.651  -6.812  1.00 37.67           C  <-- err: 3.2622
ATOM   1706  NE  ARG A 221     -20.185  -6.483  -7.949  1.00 37.67           N  <-- err: 4.7142
ATOM   1707  CZ  ARG A 221     -21.333  -7.114  -8.111  1.00 37.67           C  <-- err: 5.5418
ATOM   1708  NH1 ARG A 221     -21.636  -8.132  -7.344  1.00 37.67           N  <-- err: 5.1199
ATOM   1709  NH2 ARG A 221     -22.154  -6.751  -9.065  1.00 37.67           N  <-- err: 6.893 ***
ATOM   1710  N   PHE A 222     -19.265  -4.693  -4.950  1.00 43.40           N  <-- err: 0.2662
ATOM   1711  CA  PHE A 222     -19.062  -3.322  -5.439  1.00 43.40           C  <-- err: 0.3603
ATOM   1712  C   PHE A 222     -19.890  -2.306  -4.639  1.00 43.40           C  <-- err: 0.3793
ATOM   1713  O   PHE A 222     -20.639  -2.687  -3.740  1.00 43.40           O  <-- err: 0.3738
ATOM   1714  CB  PHE A 222     -19.216  -3.085  -6.943  1.00 43.40           C  <-- err: 0.5545
ATOM   1715  CG  PHE A 222     -18.073  -2.319  -7.564  1.00 43.40           C  <-- err: 0.6764
ATOM   1716  CD1 PHE A 222     -18.088  -0.934  -7.569  1.00 43.40           C  <-- err: 0.6106
ATOM   1717  CD2 PHE A 222     -17.105  -2.990  -8.294  1.00 43.40           C  <-- err: 0.9258
ATOM   1718  CE1 PHE A 222     -17.084  -0.233  -8.216  1.00 43.40           C  <-- err: 0.7619
ATOM   1719  CE2 PHE A 222     -16.025  -2.297  -8.815  1.00 43.40           C  <-- err: 1.0808
ATOM   1720  CZ  PHE A 222     -15.996  -0.908  -8.743  1.00 43.40           C  <-- err: 1.0543
```

```
*************** AF-Q62469-F1-model_v4.pdb max_error: 5.6124
***** original
ATOM   8826  N   LEU A1150     125.032  84.361 -33.860  1.00 65.50           N
ATOM   8827  CA  LEU A1150     126.164  84.876 -33.099  1.00 65.50           C
ATOM   8828  C   LEU A1150     126.351  84.145 -31.733  1.00 65.50           C
ATOM   8829  CB  LEU A1150     126.148  86.437 -32.915  1.00 65.50           C
ATOM   8830  O   LEU A1150     126.851  84.809 -30.850  1.00 65.50           O
ATOM   8831  CG  LEU A1150     125.699  87.427 -34.024  1.00 65.50           C
ATOM   8832  CD1 LEU A1150     125.045  88.678 -33.409  1.00 65.50           C
ATOM   8833  CD2 LEU A1150     126.873  87.964 -34.843  1.00 65.50           C
ATOM   8834  N   TRP A1151     125.981  82.847 -31.516  1.00 65.61           N
ATOM   8835  CA  TRP A1151     126.615  81.897 -30.527  1.00 65.61           C
ATOM   8836  C   TRP A1151     127.428  80.793 -31.186  1.00 65.61           C
ATOM   8837  CB  TRP A1151     125.917  81.228 -29.346  1.00 65.61           C
ATOM   8838  O   TRP A1151     128.600  80.578 -30.881  1.00 65.61           O
ATOM   8839  CG  TRP A1151     126.909  80.630 -28.328  1.00 65.61           C
ATOM   8840  CD1 TRP A1151     128.252  80.845 -28.191  1.00 65.61           C
ATOM   8841  CD2 TRP A1151     126.661  79.623 -27.309  1.00 65.61           C
ATOM   8842  CE2 TRP A1151     127.864  79.358 -26.579  1.00 65.61           C
ATOM   8843  CE3 TRP A1151     125.552  78.813 -27.016  1.00 65.61           C
ATOM   8844  NE1 TRP A1151     128.776  80.223 -27.094  1.00 65.61           N
ATOM   8845  CH2 TRP A1151     126.894  77.464 -25.473  1.00 65.61           C
ATOM   8846  CZ2 TRP A1151     127.984  78.327 -25.647  1.00 65.61           C
ATOM   8847  CZ3 TRP A1151     125.679  77.721 -26.139  1.00 65.61           C
ATOM   8848  N   LYS A1152     126.886  80.254 -32.274  1.00 66.29           N
ATOM   8849  CA  LYS A1152     127.756  79.779 -33.350  1.00 66.29           C
ATOM   8850  C   LYS A1152     128.764  80.879 -33.781  1.00 66.29           C
ATOM   8851  CB  LYS A1152     126.888  79.154 -34.455  1.00 66.29           C
ATOM   8852  O   LYS A1152     129.708  80.556 -34.492  1.00 66.29           O
ATOM   8853  CG  LYS A1152     126.312  77.812 -33.954  1.00 66.29           C
ATOM   8854  CD  LYS A1152     125.417  77.122 -34.990  1.00 66.29           C
ATOM   8855  CE  LYS A1152     125.070  75.705 -34.511  1.00 66.29           C
ATOM   8856  NZ  LYS A1152     124.219  74.983 -35.491  1.00 66.29           N
***** foldcomp
ATOM   8826  N   LEU A1150     121.770  82.308 -31.262  1.00 65.63           N  <-- err: 4.6481
ATOM   8827  CA  LEU A1150     122.856  82.790 -30.464  1.00 65.63           C  <-- err: 4.7157
ATOM   8828  C   LEU A1150     122.995  82.041 -29.084  1.00 65.63           C  <-- err: 4.7652
ATOM   8829  O   LEU A1150     123.484  82.621 -28.109  1.00 65.63           O  <-- err: 4.8618
ATOM   8830  CB  LEU A1150     122.864  84.305 -30.253  1.00 65.63           C  <-- err: 4.7346
ATOM   8831  CG  LEU A1150     122.456  85.155 -31.458  1.00 65.63           C  <-- err: 4.7184
ATOM   8832  CD1 LEU A1150     121.748  86.421 -31.004  1.00 65.63           C  <-- err: 4.6635
ATOM   8833  CD2 LEU A1150     123.684  85.612 -32.229  1.00 65.63           C  <-- err: 4.7471
ATOM   8834  N   TRP A1151     122.585  80.745 -28.827  1.00 65.63           N  <-- err: 4.8148
ATOM   8835  CA  TRP A1151     123.150  79.795 -27.830  1.00 65.63           C  <-- err: 4.8681
ATOM   8836  C   TRP A1151     123.914  78.652 -28.451  1.00 65.63           C  <-- err: 4.9409
ATOM   8837  O   TRP A1151     125.069  78.416 -28.099  1.00 65.63           O  <-- err: 4.9882
ATOM   8838  CB  TRP A1151     122.336  79.316 -26.627  1.00 65.63           C  <-- err: 4.8859
ATOM   8839  CG  TRP A1151     123.153  78.642 -25.564  1.00 65.63           C  <-- err: 5.0695
ATOM   8840  CD1 TRP A1151     124.507  78.673 -25.437  1.00 65.63           C  <-- err: 5.131
ATOM   8841  CD2 TRP A1151     122.664  77.779 -24.520  1.00 65.63           C  <-- err: 5.211
ATOM   8842  NE1 TRP A1151     124.885  78.043 -24.270  1.00 65.63           N  <-- err: 5.2789
ATOM   8843  CE2 TRP A1151     123.769  77.368 -23.747  1.00 65.63           C  <-- err: 5.3618
ATOM   8844  CE3 TRP A1151     121.414  77.222 -24.226  1.00 65.63           C  <-- err: 5.2382
ATOM   8845  CZ2 TRP A1151     123.603  76.401 -22.748  1.00 65.63           C  <-- err: 5.5953
ATOM   8846  CZ3 TRP A1151     121.315  76.191 -23.308  1.00 65.63           C  <-- err: 5.4222
ATOM   8847  CH2 TRP A1151     122.387  75.803 -22.570  1.00 65.63           C  <-- err: 5.6124 ***
ATOM   8848  N   LYS A1152     123.317  78.079 -29.494  1.00 66.34           N  <-- err: 5.0196
ATOM   8849  CA  LYS A1152     124.130  77.571 -30.528  1.00 66.34           C  <-- err: 5.0977
ATOM   8850  C   LYS A1152     125.069  78.621 -30.914  1.00 66.34           C  <-- err: 5.1934
ATOM   8851  O   LYS A1152     126.050  78.357 -31.609  1.00 66.34           O  <-- err: 5.1506
ATOM   8852  CB  LYS A1152     123.370  77.086 -31.764  1.00 66.34           C  <-- err: 4.8882
ATOM   8853  CG  LYS A1152     122.622  75.779 -31.552  1.00 66.34           C  <-- err: 4.8496
ATOM   8854  CD  LYS A1152     121.857  75.358 -32.796  1.00 66.34           C  <-- err: 4.5386
ATOM   8855  CE  LYS A1152     121.310  73.945 -32.671  1.00 66.34           C  <-- err: 4.541
ATOM   8856  NZ  LYS A1152     120.558  73.522 -33.885  1.00 66.34           N  <-- err: 4.2564
```

```
*************** AF-E9Q842-F1-model_v4.pdb max_error: 5.3668
***** original
ATOM   6690  N   THR A 898      19.907 -13.009 -38.177  1.00 32.96           N
ATOM   6691  CA  THR A 898      21.258 -12.409 -38.483  1.00 32.96           C
ATOM   6692  C   THR A 898      21.318 -11.171 -39.494  1.00 32.96           C
ATOM   6693  CB  THR A 898      22.274 -13.476 -38.905  1.00 32.96           C
ATOM   6694  O   THR A 898      20.949 -10.071 -39.103  1.00 32.96           O
ATOM   6695  CG2 THR A 898      23.248 -13.982 -37.840  1.00 32.96           C
ATOM   6696  OG1 THR A 898      21.687 -14.490 -39.685  1.00 32.96           O
ATOM   6697  N   ARG A 899      21.891 -11.208 -40.744  1.00 34.69           N
ATOM   6698  CA  ARG A 899      22.186 -10.027 -41.661  1.00 34.69           C
ATOM   6699  C   ARG A 899      21.291 -10.004 -42.934  1.00 34.69           C
ATOM   6700  CB  ARG A 899      23.623  -9.875 -42.353  1.00 34.69           C
ATOM   6701  O   ARG A 899      21.095 -11.060 -43.536  1.00 34.69           O
ATOM   6702  CG  ARG A 899      25.121  -9.565 -42.008  1.00 34.69           C
ATOM   6703  CD  ARG A 899      25.646  -8.285 -41.340  1.00 34.69           C
ATOM   6704  NE  ARG A 899      26.739  -8.588 -40.391  1.00 34.69           N
ATOM   6705  NH1 ARG A 899      27.142  -6.507 -39.464  1.00 34.69           N
ATOM   6706  NH2 ARG A 899      28.443  -8.244 -38.951  1.00 34.69           N
ATOM   6707  CZ  ARG A 899      27.429  -7.769 -39.613  1.00 34.69           C
ATOM   6708  N   ARG A 900      21.166  -8.777 -43.483  1.00 32.16           N
ATOM   6709  CA  ARG A 900      21.388  -8.335 -44.893  1.00 32.16           C
ATOM   6710  C   ARG A 900      20.227  -8.577 -45.870  1.00 32.16           C
ATOM   6711  CB  ARG A 900      22.751  -8.817 -45.493  1.00 32.16           C
ATOM   6712  O   ARG A 900      19.839  -9.724 -46.039  1.00 32.16           O
ATOM   6713  CG  ARG A 900      23.949  -7.860 -45.265  1.00 32.16           C
ATOM   6714  CD  ARG A 900      25.373  -8.418 -45.439  1.00 32.16           C
ATOM   6715  NE  ARG A 900      26.366  -7.538 -44.765  1.00 32.16           N
ATOM   6716  NH1 ARG A 900      28.285  -8.757 -45.137  1.00 32.16           N
ATOM   6717  NH2 ARG A 900      28.394  -6.889 -43.931  1.00 32.16           N
ATOM   6718  CZ  ARG A 900      27.671  -7.733 -44.615  1.00 32.16           C
***** foldcomp
ATOM   6690  N   THR A 898      19.616 -12.895 -38.216  1.00 32.85           N  <-- err: 0.315
ATOM   6691  CA  THR A 898      20.919 -12.312 -38.522  1.00 32.85           C  <-- err: 0.3548
ATOM   6692  C   THR A 898      20.973 -11.123 -39.494  1.00 32.85           C  <-- err: 0.3483
ATOM   6693  O   THR A 898      20.601 -10.008 -39.130  1.00 32.85           O  <-- err: 0.3547
ATOM   6694  CB  THR A 898      21.929 -13.381 -38.943  1.00 32.85           C  <-- err: 0.3599
ATOM   6695  OG1 THR A 898      21.263 -14.392 -39.703  1.00 32.85           O  <-- err: 0.4356
ATOM   6696  CG2 THR A 898      22.845 -13.764 -37.792  1.00 32.85           C  <-- err: 0.4607
ATOM   6697  N   ARG A 899      21.522 -11.154 -40.710  1.00 34.58           N  <-- err: 0.3745
ATOM   6698  CA  ARG A 899      21.800 -10.018 -41.594  1.00 34.58           C  <-- err: 0.3919
ATOM   6699  C   ARG A 899      20.916  -9.988 -42.846  1.00 34.58           C  <-- err: 0.3855
ATOM   6700  O   ARG A 899      20.721 -11.023 -43.481  1.00 34.58           O  <-- err: 0.3798
ATOM   6701  CB  ARG A 899      23.274  -9.864 -41.973  1.00 34.58           C  <-- err: 0.5161
ATOM   6702  CG  ARG A 899      24.155  -9.419 -40.803  1.00 34.58           C  <-- err: 1.5513
ATOM   6703  CD  ARG A 899      23.695  -8.074 -40.264  1.00 34.58           C  <-- err: 2.238
ATOM   6704  NE  ARG A 899      23.796  -8.007 -38.809  1.00 34.58           N  <-- err: 3.3914
ATOM   6705  CZ  ARG A 899      23.468  -6.963 -38.071  1.00 34.58           C  <-- err: 4.3263
ATOM   6706  NH1 ARG A 899      22.894  -5.923 -38.623  1.00 34.58           N  <-- err: 4.3696
ATOM   6707  NH2 ARG A 899      23.702  -6.971 -36.782  1.00 34.58           N  <-- err: 5.3668 ***
ATOM   6708  N   ARG A 900      20.791  -8.771 -43.392  1.00 32.27           N  <-- err: 0.3859
ATOM   6709  CA  ARG A 900      21.004  -8.335 -44.774  1.00 32.27           C  <-- err: 0.402
ATOM   6710  C   ARG A 900      19.844  -8.567 -45.747  1.00 32.27           C  <-- err: 0.4024
ATOM   6711  O   ARG A 900      19.438  -9.711 -45.950  1.00 32.27           O  <-- err: 0.411
ATOM   6712  CB  ARG A 900      22.324  -8.805 -45.389  1.00 32.27           C  <-- err: 0.4396
ATOM   6713  CG  ARG A 900      23.479  -7.828 -45.158  1.00 32.27           C  <-- err: 0.4831
ATOM   6714  CD  ARG A 900      24.817  -8.515 -45.373  1.00 32.27           C  <-- err: 0.5682
ATOM   6715  NE  ARG A 900      25.915  -7.772 -44.761  1.00 32.27           N  <-- err: 0.5081
ATOM   6716  CZ  ARG A 900      27.168  -8.182 -44.697  1.00 32.27           C  <-- err: 0.6792
ATOM   6717  NH1 ARG A 900      27.521  -9.307 -45.266  1.00 32.27           N  <-- err: 0.9502
ATOM   6718  NH2 ARG A 900      28.065  -7.457 -44.075  1.00 32.27           N  <-- err: 0.672
```

```
*************** AF-Q8VIK5-F1-model_v4.pdb max_error: 5.0432
***** original
ATOM   5469  N   SER A 753     -42.566  41.413  40.762  1.00 56.54           N
ATOM   5470  CA  SER A 753     -41.340  42.348  39.952  1.00 56.54           C
ATOM   5471  C   SER A 753     -40.384  42.245  38.410  1.00 56.54           C
ATOM   5472  CB  SER A 753     -40.248  42.484  41.019  1.00 56.54           C
ATOM   5473  O   SER A 753     -40.833  41.395  37.649  1.00 56.54           O
ATOM   5474  OG  SER A 753     -39.365  43.556  40.735  1.00 56.54           O
ATOM   5475  N   LEU A 754     -39.180  42.980  37.840  1.00 55.79           N
ATOM   5476  CA  LEU A 754     -38.332  43.149  36.366  1.00 55.79           C
ATOM   5477  C   LEU A 754     -36.707  43.071  35.878  1.00 55.79           C
ATOM   5478  CB  LEU A 754     -38.728  44.495  35.577  1.00 55.79           C
ATOM   5479  O   LEU A 754     -36.471  42.739  34.714  1.00 55.79           O
ATOM   5480  CG  LEU A 754     -38.560  44.725  33.995  1.00 55.79           C
ATOM   5481  CD1 LEU A 754     -39.611  45.743  33.508  1.00 55.79           C
ATOM   5482  CD2 LEU A 754     -37.273  45.355  33.385  1.00 55.79           C
ATOM   5483  N   GLY A 755     -35.543  43.419  36.519  1.00 62.11           N
ATOM   5484  CA  GLY A 755     -34.291  44.044  35.860  1.00 62.11           C
ATOM   5485  C   GLY A 755     -33.004  43.393  35.164  1.00 62.11           C
ATOM   5486  O   GLY A 755     -31.976  44.074  35.119  1.00 62.11           O
***** foldcomp
ATOM   5469  N   SER A 753     -45.423  41.051  43.191  1.00 56.40           N  <-- err: 3.7674
ATOM   5470  CA  SER A 753     -44.335  41.868  42.447  1.00 56.40           C  <-- err: 3.9275
ATOM   5471  C   SER A 753     -43.466  41.820  41.075  1.00 56.40           C  <-- err: 4.0965
ATOM   5472  O   SER A 753     -43.760  41.033  40.178  1.00 56.40           O  <-- err: 3.8851
ATOM   5473  CB  SER A 753     -43.408  41.989  43.658  1.00 56.40           C  <-- err: 4.1467
ATOM   5474  OG  SER A 753     -42.471  43.037  43.482  1.00 56.40           O  <-- err: 4.1788
ATOM   5475  N   LEU A 754     -42.347  42.493  40.509  1.00 55.71           N  <-- err: 4.1702
ATOM   5476  CA  LEU A 754     -41.558  42.672  39.181  1.00 55.71           C  <-- err: 4.308
ATOM   5477  C   LEU A 754     -40.020  42.635  38.661  1.00 55.71           C  <-- err: 4.3487
ATOM   5478  O   LEU A 754     -39.766  42.315  37.495  1.00 55.71           O  <-- err: 4.3325
ATOM   5479  CB  LEU A 754     -42.284  43.893  38.612  1.00 55.71           C  <-- err: 4.7137
ATOM   5480  CG  LEU A 754     -42.190  44.088  37.098  1.00 55.71           C  <-- err: 4.8178
ATOM   5481  CD1 LEU A 754     -43.414  44.823  36.576  1.00 55.71           C  <-- err: 4.9721
ATOM   5482  CD2 LEU A 754     -41.015  44.987  36.746  1.00 55.71           C  <-- err: 5.0432 ***
ATOM   5483  N   GLY A 755     -38.810  42.986  39.209  1.00 62.25           N  <-- err: 4.254
ATOM   5484  CA  GLY A 755     -37.562  43.604  38.518  1.00 62.25           C  <-- err: 4.2377
ATOM   5485  C   GLY A 755     -36.255  43.011  37.770  1.00 62.25           C  <-- err: 4.184
ATOM   5486  O   GLY A 755     -35.231  43.685  37.673  1.00 62.25           O  <-- err: 4.1556
```

5.2 PDC

```
*************** AF-Q0QWG9-F1-model_v4.pdb max_error: 13.7159
***** original
ATOM   1864  N   ARG A 242      42.664 -47.079 -13.654  1.00 36.08           N
ATOM   1865  CA  ARG A 242      43.038 -48.236 -12.742  1.00 36.08           C
ATOM   1866  C   ARG A 242      44.533 -48.691 -12.745  1.00 36.08           C
ATOM   1867  CB  ARG A 242      42.054 -49.405 -13.020  1.00 36.08           C
ATOM   1868  O   ARG A 242      45.209 -48.413 -13.719  1.00 36.08           O
ATOM   1869  CG  ARG A 242      40.600 -49.001 -12.735  1.00 36.08           C
ATOM   1870  CD  ARG A 242      39.559 -50.119 -12.803  1.00 36.08           C
ATOM   1871  NE  ARG A 242      38.201 -49.547 -12.648  1.00 36.08           N
ATOM   1872  NH1 ARG A 242      36.985 -51.257 -13.587  1.00 36.08           N
ATOM   1873  NH2 ARG A 242      35.939 -49.410 -12.908  1.00 36.08           N
ATOM   1874  CZ  ARG A 242      37.054 -50.073 -13.047  1.00 36.08           C
ATOM   1875  N   ARG A 243      45.151 -49.388 -11.754  1.00 37.56           N
ATOM   1876  CA  ARG A 243      44.944 -49.898 -10.339  1.00 37.56           C
ATOM   1877  C   ARG A 243      46.323 -50.505  -9.854  1.00 37.56           C
ATOM   1878  CB  ARG A 243      43.906 -51.045 -10.350  1.00 37.56           C
ATOM   1879  O   ARG A 243      47.205 -50.530 -10.708  1.00 37.56           O
ATOM   1880  CG  ARG A 243      42.549 -50.706  -9.712  1.00 37.56           C
ATOM   1881  CD  ARG A 243      41.635 -51.935  -9.856  1.00 37.56           C
ATOM   1882  NE  ARG A 243      40.259 -51.694  -9.373  1.00 37.56           N
ATOM   1883  NH1 ARG A 243      39.329 -53.613 -10.236  1.00 37.56           N
ATOM   1884  NH2 ARG A 243      38.058 -52.211  -9.058  1.00 37.56           N
ATOM   1885  CZ  ARG A 243      39.226 -52.503  -9.557  1.00 37.56           C
ATOM   1886  N   PRO A 244      46.515 -51.171  -8.677  1.00 40.93           N
ATOM   1887  CA  PRO A 244      46.100 -50.967  -7.267  1.00 40.93           C
ATOM   1888  C   PRO A 244      47.323 -51.050  -6.266  1.00 40.93           C
ATOM   1889  CB  PRO A 244      45.119 -52.132  -7.055  1.00 40.93           C
ATOM   1890  O   PRO A 244      48.431 -50.729  -6.676  1.00 40.93           O
ATOM   1891  CG  PRO A 244      45.753 -53.278  -7.856  1.00 40.93           C
ATOM   1892  CD  PRO A 244      46.801 -52.601  -8.750  1.00 40.93           C
***** pdc
ATOM   1864  N   ARG A 242      42.753 -47.034 -13.604  1.00 36.16           N  <-- err: 0.1116
ATOM   1865  CA  ARG A 242      42.956 -48.214 -12.768  1.00 36.16           C  <-- err: 0.0888
ATOM   1866  C   ARG A 242      44.400 -48.312 -12.305  1.00 36.16           C  <-- err: 0.5958
ATOM   1867  CB  ARG A 242      42.576 -49.479 -13.519  1.00 36.16           C  <-- err: 0.7259
ATOM   1868  O   ARG A 242      45.293 -47.729 -12.918  1.00 36.16           O  <-- err: 1.0567
ATOM   1869  CG  ARG A 242      41.095 -49.814 -13.447  1.00 36.16           C  <-- err: 1.1887
ATOM   1870  CD  ARG A 242      40.819 -51.247 -13.869  1.00 36.16           C  <-- err: 1.9991
ATOM   1871  NE  ARG A 242      39.422 -51.438 -14.247  1.00 36.16           N  <-- err: 2.7611
ATOM   1872  NH1 ARG A 242      39.801 -53.349 -15.488  1.00 36.16           N  <-- err: 3.99
ATOM   1873  NH2 ARG A 242      37.696 -52.455 -15.397  1.00 36.16           N  <-- err: 4.3075
ATOM   1874  CZ  ARG A 242      38.976 -52.408 -15.039  1.00 36.16           C  <-- err: 3.6214
ATOM   1875  N   ARG A 243      43.821 -48.925 -10.890  1.00 37.56           N  <-- err: 1.6522
ATOM   1876  CA  ARG A 243      44.856 -49.814 -10.368  1.00 37.56           C  <-- err: 0.1251
ATOM   1877  C   ARG A 243      44.776 -49.910  -8.853  1.00 37.56           C  <-- err: 1.9363
ATOM   1878  CB  ARG A 243      46.238 -49.328 -10.773  1.00 37.56           C  <-- err: 2.9266
ATOM   1879  O   ARG A 243      43.843 -49.391  -8.242  1.00 37.56           O  <-- err: 4.3222
ATOM   1880  CG  ARG A 243      47.102 -50.400 -11.415  1.00 37.56           C  <-- err: 4.8707
ATOM   1881  CD  ARG A 243      48.369 -49.816 -12.017  1.00 37.56           C  <-- err: 7.3829
ATOM   1882  NE  ARG A 243      49.081 -50.796 -12.830  1.00 37.56           N  <-- err: 9.5176
ATOM   1883  NH1 ARG A 243      50.569 -49.274 -13.727  1.00 37.56           N  <-- err: 12.544
ATOM   1884  NH2 ARG A 243      50.699 -51.478 -14.330  1.00 37.56           N  <-- err: 13.7159 ***
ATOM   1885  CZ  ARG A 243      50.110 -50.518 -13.624  1.00 37.56           C  <-- err: 11.7874
ATOM   1886  N   PRO A 244      46.244 -50.572  -8.674  1.00 40.86           N  <-- err: 0.6575
ATOM   1887  CA  PRO A 244      46.056 -50.914  -7.268  1.00 40.86           C  <-- err: 0.0689
ATOM   1888  C   PRO A 244      47.346 -50.730  -6.485  1.00 40.86           C  <-- err: 0.3884
ATOM   1889  CB  PRO A 244      45.573 -52.347  -7.121  1.00 40.86           C  <-- err: 0.5067
ATOM   1890  O   PRO A 244      48.157 -49.866  -6.813  1.00 40.86           O  <-- err: 0.9158
ATOM   1891  CG  PRO A 244      46.098 -53.036  -8.332  1.00 40.86           C  <-- err: 0.6357
ATOM   1892  CD  PRO A 244      47.039 -52.052  -8.961  1.00 40.86           C  <-- err: 0.6345
```

```
*************** AF-Q7TNC6-F1-model_v4.pdb max_error: 13.6139
***** original
ATOM  11345  N   ASN A1529      57.117  25.585  83.985  1.00 28.60           N
ATOM  11346  CA  ASN A1529      58.113  26.680  84.173  1.00 28.60           C
ATOM  11347  C   ASN A1529      58.237  27.681  82.986  1.00 28.60           C
ATOM  11348  CB  ASN A1529      59.507  26.148  84.583  1.00 28.60           C
ATOM  11349  O   ASN A1529      58.435  27.243  81.864  1.00 28.60           O
ATOM  11350  CG  ASN A1529      59.620  25.750  86.042  1.00 28.60           C
ATOM  11351  ND2 ASN A1529      60.435  24.771  86.355  1.00 28.60           N
ATOM  11352  OD1 ASN A1529      59.035  26.351  86.928  1.00 28.60           O
ATOM  11353  N   ARG A1530      58.263  29.022  83.169  1.00 30.38           N
ATOM  11354  CA  ARG A1530      58.026  29.892  84.361  1.00 30.38           C
ATOM  11355  C   ARG A1530      58.020  31.400  83.972  1.00 30.38           C
ATOM  11356  CB  ARG A1530      59.191  29.710  85.364  1.00 30.38           C
ATOM  11357  O   ARG A1530      58.741  31.754  83.052  1.00 30.38           O
ATOM  11358  CG  ARG A1530      58.771  29.627  86.837  1.00 30.38           C
ATOM  11359  CD  ARG A1530      60.037  29.455  87.690  1.00 30.38           C
ATOM  11360  NE  ARG A1530      59.782  29.636  89.131  1.00 30.38           N
ATOM  11361  NH1 ARG A1530      61.837  28.924  89.879  1.00 30.38           N
ATOM  11362  NH2 ARG A1530      60.313  29.618  91.348  1.00 30.38           N
ATOM  11363  CZ  ARG A1530      60.640  29.390  90.108  1.00 30.38           C
ATOM  11364  N   ASN A1531      57.422  32.267  84.815  1.00 28.67           N
ATOM  11365  CA  ASN A1531      57.756  33.710  85.021  1.00 28.67           C
ATOM  11366  C   ASN A1531      57.508  34.735  83.868  1.00 28.67           C
ATOM  11367  CB  ASN A1531      59.223  33.764  85.511  1.00 28.67           C
ATOM  11368  O   ASN A1531      57.392  34.337  82.722  1.00 28.67           O
ATOM  11369  CG  ASN A1531      59.454  33.187  86.896  1.00 28.67           C
ATOM  11370  ND2 ASN A1531      60.697  32.999  87.268  1.00 28.67           N
ATOM  11371  OD1 ASN A1531      58.550  32.909  87.668  1.00 28.67           O
***** pdc
ATOM  11345  N   ASN A1529      57.297  25.383  83.937  1.00 28.67           N  <-- err: 0.2748
ATOM  11346  CA  ASN A1529      58.043  26.625  84.123  1.00 28.67           C  <-- err: 0.1021
ATOM  11347  C   ASN A1529      57.437  27.751  83.301  1.00 28.67           C  <-- err: 0.8626
ATOM  11348  CB  ASN A1529      59.501  26.440  83.735  1.00 28.67           C  <-- err: 0.8969
ATOM  11349  O   ASN A1529      57.139  27.572  82.121  1.00 28.67           O  <-- err: 1.3616
ATOM  11350  CG  ASN A1529      60.347  25.907  84.879  1.00 28.67           C  <-- err: 1.3805
ATOM  11351  ND2 ASN A1529      61.272  25.124  84.667  1.00 28.67           N  <-- err: 1.9169
ATOM  11352  OD1 ASN A1529      60.029  26.331  86.098  1.00 28.67           O  <-- err: 1.2951
ATOM  11353  N   ARG A1530      57.566  28.527  84.792  1.00 30.37           N  <-- err: 1.8344
ATOM  11354  CA  ARG A1530      58.043  29.825  84.323  1.00 30.37           C  <-- err: 0.0789
ATOM  11355  C   ARG A1530      57.355  30.959  85.065  1.00 30.37           C  <-- err: 1.3533
ATOM  11356  CB  ARG A1530      57.804  29.978  82.829  1.00 30.37           C  <-- err: 2.902
ATOM  11357  O   ARG A1530      56.291  30.766  85.652  1.00 30.37           O  <-- err: 3.7066
ATOM  11358  CG  ARG A1530      59.064  30.274  82.032  1.00 30.37           C  <-- err: 4.8572
ATOM  11359  CD  ARG A1530      58.857  30.044  80.543  1.00 30.37           C  <-- err: 7.2677
ATOM  11360  NE  ARG A1530      59.981  30.545  79.758  1.00 30.37           N  <-- err: 9.4191
ATOM  11361  NH1 ARG A1530      59.260  29.630  77.760  1.00 30.37           N  <-- err: 12.4101
ATOM  11362  NH2 ARG A1530      61.206  30.834  77.818  1.00 30.37           N  <-- err: 13.6139 ***
ATOM  11363  CZ  ARG A1530      60.148  30.338  78.454  1.00 30.37           C  <-- err: 11.7028
ATOM  11364  N   ASN A1531      57.465  32.217  84.839  1.00 28.67           N  <-- err: 0.0702
ATOM  11365  CA  ASN A1531      57.843  33.625  84.923  1.00 28.67           C  <-- err: 0.1562
ATOM  11366  C   ASN A1531      56.713  34.521  84.442  1.00 28.67           C  <-- err: 1.0036
ATOM  11367  CB  ASN A1531      59.092  33.897  84.100  1.00 28.67           C  <-- err: 1.4233
ATOM  11368  O   ASN A1531      55.766  34.049  83.815  1.00 28.67           O  <-- err: 1.9803
ATOM  11369  CG  ASN A1531      60.371  33.703  84.897  1.00 28.67           C  <-- err: 2.259
ATOM  11370  ND2 ASN A1531      61.470  33.756  84.348  1.00 28.67           N  <-- err: 3.114
ATOM  11371  OD1 ASN A1531      60.229  33.476  86.199  1.00 28.67           O  <-- err: 2.3018
```

```
*************** AF-P23116-F1-model_v4.pdb max_error: 13.5329
***** original
ATOM   7837  N   HIS A 929     -12.870 -51.384 -15.630  1.00 29.88           N
ATOM   7838  CA  HIS A 929     -14.184 -51.161 -15.012  1.00 29.88           C
ATOM   7839  C   HIS A 929     -14.391 -49.639 -14.847  1.00 29.88           C
ATOM   7840  CB  HIS A 929     -14.346 -51.886 -13.651  1.00 29.88           C
ATOM   7841  O   HIS A 929     -13.523 -48.968 -14.305  1.00 29.88           O
ATOM   7842  CG  HIS A 929     -15.131 -53.189 -13.686  1.00 29.88           C
ATOM   7843  CD2 HIS A 929     -15.866 -53.729 -12.665  1.00 29.88           C
ATOM   7844  ND1 HIS A 929     -15.143 -54.118 -14.699  1.00 29.88           N
ATOM   7845  CE1 HIS A 929     -15.840 -55.190 -14.300  1.00 29.88           C
ATOM   7846  NE2 HIS A 929     -16.347 -54.984 -13.075  1.00 29.88           N
ATOM   7847  N   ARG A 930     -15.518 -49.054 -15.263  1.00 29.18           N
ATOM   7848  CA  ARG A 930     -16.607 -49.599 -16.094  1.00 29.18           C
ATOM   7849  C   ARG A 930     -17.448 -48.428 -16.646  1.00 29.18           C
ATOM   7850  CB  ARG A 930     -17.487 -50.545 -15.236  1.00 29.18           C
ATOM   7851  O   ARG A 930     -17.361 -47.333 -16.106  1.00 29.18           O
ATOM   7852  CG  ARG A 930     -17.771 -51.884 -15.936  1.00 29.18           C
ATOM   7853  CD  ARG A 930     -18.634 -52.811 -15.074  1.00 29.18           C
ATOM   7854  NE  ARG A 930     -18.873 -54.103 -15.752  1.00 29.18           N
ATOM   7855  NH1 ARG A 930     -19.477 -55.320 -13.903  1.00 29.18           N
ATOM   7856  NH2 ARG A 930     -19.516 -56.284 -15.910  1.00 29.18           N
ATOM   7857  CZ  ARG A 930     -19.288 -55.223 -15.187  1.00 29.18           C
ATOM   7858  N   ARG A 931     -18.239 -48.713 -17.687  1.00 29.75           N
ATOM   7859  CA  ARG A 931     -19.685 -48.424 -17.817  1.00 29.75           C
ATOM   7860  C   ARG A 931     -20.293 -47.338 -16.904  1.00 29.75           C
ATOM   7861  CB  ARG A 931     -20.426 -49.734 -17.503  1.00 29.75           C
ATOM   7862  O   ARG A 931     -20.110 -47.414 -15.693  1.00 29.75           O
ATOM   7863  CG  ARG A 931     -20.313 -50.826 -18.584  1.00 29.75           C
ATOM   7864  CD  ARG A 931     -21.273 -51.973 -18.248  1.00 29.75           C
ATOM   7865  NE  ARG A 931     -21.736 -52.668 -19.465  1.00 29.75           N
ATOM   7866  NH1 ARG A 931     -23.962 -52.059 -19.339  1.00 29.75           N
ATOM   7867  NH2 ARG A 931     -23.271 -53.319 -21.016  1.00 29.75           N
ATOM   7868  CZ  ARG A 931     -22.982 -52.675 -19.923  1.00 29.75           C
***** pdc
ATOM   7837  N   HIS A 929     -13.095 -51.486 -16.078  1.00 29.82           N  <-- err: 0.5116
ATOM   7838  CA  HIS A 929     -14.090 -51.243 -15.038  1.00 29.82           C  <-- err: 0.1274
ATOM   7839  C   HIS A 929     -14.998 -50.084 -15.413  1.00 29.82           C  <-- err: 0.9417
ATOM   7840  CB  HIS A 929     -13.416 -50.948 -13.707  1.00 29.82           C  <-- err: 1.3221
ATOM   7841  O   HIS A 929     -14.523 -49.015 -15.794  1.00 29.82           O  <-- err: 1.7942
ATOM   7842  CG  HIS A 929     -13.753 -51.925 -12.634  1.00 29.82           C  <-- err: 2.1455
ATOM   7843  CD2 HIS A 929     -13.853 -51.762 -11.297  1.00 29.82           C  <-- err: 3.1293
ATOM   7844  ND1 HIS A 929     -14.033 -53.251 -12.890  1.00 29.82           N  <-- err: 2.2927
ATOM   7845  CE1 HIS A 929     -14.290 -53.853 -11.744  1.00 29.82           C  <-- err: 3.2746
ATOM   7846  NE2 HIS A 929     -14.184 -52.948 -10.744  1.00 29.82           N  <-- err: 3.7759
ATOM   7847  N   ARG A 930     -15.531 -50.641 -16.160  1.00 29.22           N  <-- err: 1.823
ATOM   7848  CA  ARG A 930     -16.590 -49.643 -16.038  1.00 29.22           C  <-- err: 0.0732
ATOM   7849  C   ARG A 930     -17.509 -49.672 -17.247  1.00 29.22           C  <-- err: 1.3829
ATOM   7850  CB  ARG A 930     -16.001 -48.250 -15.881  1.00 29.22           C  <-- err: 2.8091
ATOM   7851  O   ARG A 930     -17.155 -50.225 -18.287  1.00 29.22           O  <-- err: 3.6281
ATOM   7852  CG  ARG A 930     -16.432 -47.540 -14.607  1.00 29.22           C  <-- err: 4.736
ATOM   7853  CD  ARG A 930     -15.686 -46.230 -14.411  1.00 29.22           C  <-- err: 7.2415
ATOM   7854  NE  ARG A 930     -16.028 -45.599 -13.139  1.00 29.22           N  <-- err: 9.3402
ATOM   7855  NH1 ARG A 930     -14.189 -44.198 -13.101  1.00 29.22           N  <-- err: 12.3412
ATOM   7856  NH2 ARG A 930     -15.720 -44.101 -11.404  1.00 29.22           N  <-- err: 13.5329 ***
ATOM   7857  CZ  ARG A 930     -15.317 -44.639 -12.552  1.00 29.22           C  <-- err: 11.6075
ATOM   7858  N   ARG A 931     -18.266 -48.689 -17.173  1.00 29.72           N  <-- err: 0.5153
ATOM   7859  CA  ARG A 931     -19.590 -48.443 -17.738  1.00 29.72           C  <-- err: 0.125
ATOM   7860  C   ARG A 931     -20.094 -47.060 -17.359  1.00 29.72           C  <-- err: 0.5691
ATOM   7861  CB  ARG A 931     -20.581 -49.492 -17.263  1.00 29.72           C  <-- err: 0.3744
ATOM   7862  O   ARG A 931     -19.944 -46.632 -16.215  1.00 29.72           O  <-- err: 0.9548
ATOM   7863  CG  ARG A 931     -20.601 -50.749 -18.117  1.00 29.72           C  <-- err: 0.554
ATOM   7864  CD  ARG A 931     -21.631 -51.751 -17.621  1.00 29.72           C  <-- err: 0.7554
ATOM   7865  NE  ARG A 931     -22.207 -52.520 -18.719  1.00 29.72           N  <-- err: 0.8946
ATOM   7866  NH1 ARG A 931     -24.314 -51.589 -18.536  1.00 29.72           N  <-- err: 0.9948
ATOM   7867  NH2 ARG A 931     -23.907 -53.169 -20.142  1.00 29.72           N  <-- err: 1.0913
ATOM   7868  CZ  ARG A 931     -23.468 -52.427 -19.130  1.00 29.72           C  <-- err: 0.9626
```

```
 *************** AF-Q91YE5-F1-model_v4.pdb max_error: 13.5006
***** original
ATOM  13534  N   ARG A1746     -75.953  26.967   5.549  1.00 37.53           N
ATOM  13535  CA  ARG A1746     -76.885  26.039   4.803  1.00 37.53           C
ATOM  13536  C   ARG A1746     -76.505  25.538   3.375  1.00 37.53           C
ATOM  13537  CB  ARG A1746     -78.294  26.694   4.794  1.00 37.53           C
ATOM  13538  O   ARG A1746     -75.901  26.267   2.616  1.00 37.53           O
ATOM  13539  CG  ARG A1746     -78.989  26.649   6.168  1.00 37.53           C
ATOM  13540  CD  ARG A1746     -80.340  27.380   6.152  1.00 37.53           C
ATOM  13541  NE  ARG A1746     -80.872  27.570   7.522  1.00 37.53           N
ATOM  13542  NH1 ARG A1746     -83.124  27.579   7.038  1.00 37.53           N
ATOM  13543  NH2 ARG A1746     -82.443  27.978   9.122  1.00 37.53           N
ATOM  13544  CZ  ARG A1746     -82.139  27.703   7.884  1.00 37.53           C
ATOM  13545  N   ARG A1747     -76.915  24.333   2.898  1.00 36.86           N
ATOM  13546  CA  ARG A1747     -77.370  23.053   3.538  1.00 36.86           C
ATOM  13547  C   ARG A1747     -77.539  21.881   2.514  1.00 36.86           C
ATOM  13548  CB  ARG A1747     -78.763  23.221   4.204  1.00 36.86           C
ATOM  13549  O   ARG A1747     -78.209  22.079   1.518  1.00 36.86           O
ATOM  13550  CG  ARG A1747     -78.828  22.802   5.680  1.00 36.86           C
ATOM  13551  CD  ARG A1747     -80.289  22.809   6.167  1.00 36.86           C
ATOM  13552  NE  ARG A1747     -80.387  22.748   7.641  1.00 36.86           N
ATOM  13553  NH1 ARG A1747     -82.677  22.564   7.805  1.00 36.86           N
ATOM  13554  NH2 ARG A1747     -81.452  22.733   9.658  1.00 36.86           N
ATOM  13555  CZ  ARG A1747     -81.500  22.680   8.356  1.00 36.86           C
ATOM  13556  N   MET A1748     -77.153  20.653   2.915  1.00 33.38           N
ATOM  13557  CA  MET A1748     -77.935  19.375   2.864  1.00 33.38           C
ATOM  13558  C   MET A1748     -78.433  18.669   1.556  1.00 33.38           C
ATOM  13559  CB  MET A1748     -79.142  19.540   3.815  1.00 33.38           C
ATOM  13560  O   MET A1748     -79.022  19.294   0.689  1.00 33.38           O
ATOM  13561  CG  MET A1748     -78.860  19.143   5.265  1.00 33.38           C
ATOM  13562  SD  MET A1748     -78.723  17.364   5.564  1.00 33.38           S
ATOM  13563  CE  MET A1748     -78.552  17.376   7.367  1.00 33.38           C
***** pdc
ATOM  13534  N   ARG A1746     -76.766  26.959   5.997  1.00 37.57           N  <-- err: 0.9283
ATOM  13535  CA  ARG A1746     -76.817  26.109   4.812  1.00 37.57           C  <-- err: 0.098
ATOM  13536  C   ARG A1746     -76.546  24.657   5.171  1.00 37.57           C  <-- err: 2.0009
ATOM  13537  CB  ARG A1746     -75.808  26.571   3.773  1.00 37.57           C  <-- err: 2.6903
ATOM  13538  O   ARG A1746     -75.823  24.374   6.125  1.00 37.57           O  <-- err: 3.9878
ATOM  13539  CG  ARG A1746     -76.083  27.962   3.225  1.00 37.57           C  <-- err: 4.3394
ATOM  13540  CD  ARG A1746     -75.531  28.133   1.818  1.00 37.57           C  <-- err: 6.5174
ATOM  13541  NE  ARG A1746     -75.181  29.523   1.540  1.00 37.57           N  <-- err: 8.4845
ATOM  13542  NH1 ARG A1746     -75.596  29.394  -0.729  1.00 37.57           N  <-- err: 10.9677
ATOM  13543  NH2 ARG A1746     -74.729  31.318   0.156  1.00 37.57           N  <-- err: 12.2903
ATOM  13544  CZ  ARG A1746     -75.169  30.075   0.330  1.00 37.57           C  <-- err: 10.5485
ATOM  13545  N   ARG A1747     -76.944  24.000   4.706  1.00 36.87           N  <-- err: 1.8386
ATOM  13546  CA  ARG A1747     -77.317  23.109   3.612  1.00 36.87           C  <-- err: 0.1069
ATOM  13547  C   ARG A1747     -77.100  21.654   3.996  1.00 36.87           C  <-- err: 1.5622
ATOM  13548  CB  ARG A1747     -76.515  23.427   2.360  1.00 36.87           C  <-- err: 2.9148
ATOM  13549  O   ARG A1747     -76.126  21.325   4.670  1.00 36.87           O  <-- err: 3.8526
ATOM  13550  CG  ARG A1747     -76.947  24.706   1.661  1.00 36.87           C  <-- err: 4.8286
ATOM  13551  CD  ARG A1747     -77.065  24.514   0.157  1.00 36.87           C  <-- err: 7.03
ATOM  13552  NE  ARG A1747     -77.150  25.791  -0.544  1.00 36.87           N  <-- err: 9.313
ATOM  13553  NH1 ARG A1747     -77.553  24.860  -2.620  1.00 36.87           N  <-- err: 11.8409
ATOM  13554  NH2 ARG A1747     -77.320  27.128  -2.420  1.00 36.87           N  <-- err: 13.5006 ***
ATOM  13555  CZ  ARG A1747     -77.340  25.926  -1.853  1.00 36.87           C  <-- err: 11.492
ATOM  13556  N   MET A1748     -77.550  20.812   2.746  1.00 33.47           N  <-- err: 0.4598
ATOM  13557  CA  MET A1748     -77.917  19.409   2.912  1.00 33.47           C  <-- err: 0.0615
ATOM  13558  C   MET A1748     -78.908  18.975   1.844  1.00 33.47           C  <-- err: 0.6342
ATOM  13559  CB  MET A1748     -78.515  19.167   4.288  1.00 33.47           C  <-- err: 0.8695
ATOM  13560  O   MET A1748     -79.748  19.764   1.414  1.00 33.47           O  <-- err: 1.1285
ATOM  13561  CG  MET A1748     -78.283  20.309   5.263  1.00 33.47           C  <-- err: 1.301
ATOM  13562  SD  MET A1748     -78.024  19.736   6.960  1.00 33.47           S  <-- err: 2.8397
ATOM  13563  CE  MET A1748     -77.063  21.093   7.623  1.00 33.47           C  <-- err: 4.0123
```

```
    *************** AF-Q7TSC1-F1-model_v4.pdb max_error: 13.4792
***** original
ATOM   1813  N   TYR A 246      54.905   8.646 -15.834  1.00 41.30           N
ATOM   1814  CA  TYR A 246      54.893  10.150 -15.736  1.00 41.30           C
ATOM   1815  C   TYR A 246      54.682  11.006 -17.028  1.00 41.30           C
ATOM   1816  CB  TYR A 246      56.174  10.536 -14.967  1.00 41.30           C
ATOM   1817  O   TYR A 246      54.617  10.446 -18.110  1.00 41.30           O
ATOM   1818  CG  TYR A 246      56.243  10.197 -13.478  1.00 41.30           C
ATOM   1819  CD1 TYR A 246      55.088   9.957 -12.696  1.00 41.30           C
ATOM   1820  CD2 TYR A 246      57.508  10.167 -12.857  1.00 41.30           C
ATOM   1821  CE1 TYR A 246      55.205   9.690 -11.320  1.00 41.30           C
ATOM   1822  CE2 TYR A 246      57.631   9.871 -11.486  1.00 41.30           C
ATOM   1823  OH  TYR A 246      56.557   9.453  -9.376  1.00 41.30           O
ATOM   1824  CZ  TYR A 246      56.476   9.630 -10.716  1.00 41.30           C
ATOM   1825  N   ARG A 247      54.526  12.360 -17.075  1.00 39.68           N
ATOM   1826  CA  ARG A 247      54.052  13.539 -16.245  1.00 39.68           C
ATOM   1827  C   ARG A 247      53.725  14.687 -17.270  1.00 39.68           C
ATOM   1828  CB  ARG A 247      55.120  14.009 -15.226  1.00 39.68           C
ATOM   1829  O   ARG A 247      53.884  14.440 -18.457  1.00 39.68           O
ATOM   1830  CG  ARG A 247      54.834  13.502 -13.798  1.00 39.68           C
ATOM   1831  CD  ARG A 247      56.043  13.676 -12.859  1.00 39.68           C
ATOM   1832  NE  ARG A 247      55.891  12.896 -11.608  1.00 39.68           N
ATOM   1833  NH1 ARG A 247      56.293  14.541 -10.045  1.00 39.68           N
ATOM   1834  NH2 ARG A 247      55.910  12.463  -9.364  1.00 39.68           N
ATOM   1835  CZ  ARG A 247      56.029  13.302 -10.355  1.00 39.68           C
ATOM   1836  N   GLY A 248      53.281  15.936 -17.020  1.00 43.53           N
ATOM   1837  CA  GLY A 248      52.934  16.814 -15.871  1.00 43.53           C
ATOM   1838  C   GLY A 248      52.600  18.254 -16.398  1.00 43.53           C
ATOM   1839  O   GLY A 248      52.796  18.485 -17.587  1.00 43.53           O
***** pdc
ATOM   1813  N   TYR A 246      55.235   8.792 -16.273  1.00 41.38           N  <-- err: 0.5683
ATOM   1814  CA  TYR A 246      54.909  10.119 -15.759  1.00 41.38           C  <-- err: 0.0418
ATOM   1815  C   TYR A 246      55.138  11.185 -16.817  1.00 41.38           C  <-- err: 0.5334
ATOM   1816  CB  TYR A 246      55.740  10.439 -14.527  1.00 41.38           C  <-- err: 0.6256
ATOM   1817  O   TYR A 246      55.668  10.896 -17.889  1.00 41.38           O  <-- err: 1.1644
ATOM   1818  CG  TYR A 246      55.865   9.285 -13.560  1.00 41.38           C  <-- err: 0.9906
ATOM   1819  CD1 TYR A 246      54.741   8.732 -12.956  1.00 41.38           C  <-- err: 1.2995
ATOM   1820  CD2 TYR A 246      57.108   8.746 -13.250  1.00 41.38           C  <-- err: 1.5276
ATOM   1821  CE1 TYR A 246      54.879   7.670 -12.069  1.00 41.38           C  <-- err: 2.1789
ATOM   1822  CE2 TYR A 246      57.202   7.684 -12.357  1.00 41.38           C  <-- err: 2.3928
ATOM   1823  OH  TYR A 246      56.140   7.160 -11.785  1.00 41.38           O  <-- err: 3.3519
ATOM   1824  CZ  TYR A 246      56.271   6.098 -10.897  1.00 41.38           C  <-- err: 3.5426
ATOM   1825  N   ARG A 247      53.933  12.192 -15.575  1.00 39.68           N  <-- err: 1.6217
ATOM   1826  CA  ARG A 247      54.109  13.519 -16.159  1.00 39.68           C  <-- err: 0.1051
ATOM   1827  C   ARG A 247      53.263  14.551 -15.430  1.00 39.68           C  <-- err: 1.902
ATOM   1828  CB  ARG A 247      53.741  13.511 -17.633  1.00 39.68           C  <-- err: 2.8184
ATOM   1829  O   ARG A 247      52.573  14.224 -14.466  1.00 39.68           O  <-- err: 4.2064
ATOM   1830  CG  ARG A 247      54.902  13.173 -18.554  1.00 39.68           C  <-- err: 4.7679
ATOM   1831  CD  ARG A 247      54.707  13.755 -19.944  1.00 39.68           C  <-- err: 7.2103
ATOM   1832  NE  ARG A 247      55.322  12.918 -20.970  1.00 39.68           N  <-- err: 9.3793
ATOM   1833  NH1 ARG A 247      56.580  14.619 -21.897  1.00 39.68           N  <-- err: 11.8557
ATOM   1834  NH2 ARG A 247      56.645  12.527 -22.823  1.00 39.68           N  <-- err: 13.4792 ***
ATOM   1835  CZ  ARG A 247      56.177  13.352 -21.891  1.00 39.68           C  <-- err: 11.5371
ATOM   1836  N   GLY A 248      53.401  15.566 -16.765  1.00 43.48           N  <-- err: 0.4651
ATOM   1837  CA  GLY A 248      53.009  16.719 -15.959  1.00 43.48           C  <-- err: 0.1496
ATOM   1838  C   GLY A 248      52.706  17.921 -16.838  1.00 43.48           C  <-- err: 0.5619
ATOM   1839  O   GLY A 248      52.952  17.892 -18.042  1.00 43.48           O  <-- err: 0.7636
```

5.3 ProteStAr 10/10

```
*************** AF-Q80UK7-F1-model_v4.pdb max_error: 0.0087
***** original
ATOM   3202  N   LEU A 397     183.536  25.461-140.103  1.00 72.77           N
ATOM   3203  CA  LEU A 397     184.771  24.708-140.294  1.00 72.77           C
ATOM   3204  C   LEU A 397     184.811  24.048-141.677  1.00 72.77           C
ATOM   3205  CB  LEU A 397     184.915  23.677-139.160  1.00 72.77           C
ATOM   3206  O   LEU A 397     185.841  24.102-142.333  1.00 72.77           O
ATOM   3207  CG  LEU A 397     186.365  23.227-138.923  1.00 72.77           C
ATOM   3208  CD1 LEU A 397     187.150  24.301-138.158  1.00 72.77           C
ATOM   3209  CD2 LEU A 397     186.386  21.944-138.096  1.00 72.77           C
ATOM   3210  N   MET A 398     183.687  23.521-142.170  1.00 73.23           N
ATOM   3211  CA  MET A 398     183.537  22.981-143.527  1.00 73.23           C
ATOM   3212  C   MET A 398     183.739  24.075-144.581  1.00 73.23           C
ATOM   3213  CB  MET A 398     182.139  22.352-143.675  1.00 73.23           C
ATOM   3214  O   MET A 398     184.390  23.844-145.599  1.00 73.23           O
ATOM   3215  CG  MET A 398     182.020  21.402-144.867  1.00 73.23           C
ATOM   3216  SD  MET A 398     182.696  19.762-144.501  1.00 73.23           S
ATOM   3217  CE  MET A 398     182.265  18.859-146.009  1.00 73.23           C
ATOM   3218  N   GLY A 399     183.228  25.284-144.325  1.00 74.64           N
ATOM   3219  CA  GLY A 399     183.485  26.464-145.151  1.00 74.64           C
ATOM   3220  C   GLY A 399     184.970  26.832-145.179  1.00 74.64           C
ATOM   3221  O   GLY A 399     185.552  26.969-146.256  1.00 74.64           O
***** protestar_10_10
ATOM   3202  N   LEU A 397     183.535  25.465-140.107  1.00 72.77           N  <-- err: 0.0057
ATOM   3203  CA  LEU A 397     184.767  24.706-140.294  1.00 72.77           C  <-- err: 0.0045
ATOM   3204  C   LEU A 397     184.811  24.046-141.680  1.00 72.77           C  <-- err: 0.0036
ATOM   3205  CB  LEU A 397     184.910  23.672-139.161  1.00 72.77           C  <-- err: 0.0071
ATOM   3206  O   LEU A 397     185.845  24.101-142.329  1.00 72.77           O  <-- err: 0.0057
ATOM   3207  CG  LEU A 397     186.362  23.232-138.919  1.00 72.77           C  <-- err: 0.0071
ATOM   3208  CD1 LEU A 397     187.154  24.299-138.160  1.00 72.77           C  <-- err: 0.0049
ATOM   3209  CD2 LEU A 397     186.384  21.945-138.094  1.00 72.77           C  <-- err: 0.003
ATOM   3210  N   MET A 398     183.689  23.518-142.175  1.00 73.23           N  <-- err: 0.0062
ATOM   3211  CA  MET A 398     183.535  22.979-143.528  1.00 73.23           C  <-- err: 0.003
ATOM   3212  C   MET A 398     183.744  24.079-144.584  1.00 73.23           C  <-- err: 0.0071
ATOM   3213  CB  MET A 398     182.138  22.352-143.671  1.00 73.23           C  <-- err: 0.0041
ATOM   3214  O   MET A 398     184.393  23.848-145.596  1.00 73.23           O  <-- err: 0.0058
ATOM   3215  CG  MET A 398     182.017  21.406-144.870  1.00 73.23           C  <-- err: 0.0058
ATOM   3216  SD  MET A 398     182.699  19.767-144.496  1.00 73.23           S  <-- err: 0.0077
ATOM   3217  CE  MET A 398     182.270  18.854-146.014  1.00 73.23           C  <-- err: 0.0087 ***
ATOM   3218  N   GLY A 399     183.227  25.289-144.320  1.00 74.64           N  <-- err: 0.0071
ATOM   3219  CA  GLY A 399     183.480  26.466-145.156  1.00 74.64           C  <-- err: 0.0073
ATOM   3220  C   GLY A 399     184.965  26.829-145.178  1.00 74.64           C  <-- err: 0.0059
ATOM   3221  O   GLY A 399     185.548  26.972-146.256  1.00 74.64           O  <-- err: 0.005
```

```
*************** AF-Q7TME2-F1-model_v4.pdb max_error: 0.0087
***** original
ATOM   6474  N   ALA A 834     219.304 -21.049-169.746  1.00 77.18           N
ATOM   6475  CA  ALA A 834     220.532 -20.254-169.740  1.00 77.18           C
ATOM   6476  C   ALA A 834     221.783 -21.113-169.473  1.00 77.18           C
ATOM   6477  CB  ALA A 834     220.375 -19.128-168.710  1.00 77.18           C
ATOM   6478  O   ALA A 834     222.795 -20.964-170.167  1.00 77.18           O
ATOM   6479  N   LEU A 835     221.705 -22.052-168.523  1.00 79.00           N
ATOM   6480  CA  LEU A 835     222.758 -23.034-168.244  1.00 79.00           C
ATOM   6481  C   LEU A 835     223.068 -23.896-169.471  1.00 79.00           C
ATOM   6482  CB  LEU A 835     222.322 -23.919-167.059  1.00 79.00           C
ATOM   6483  O   LEU A 835     224.232 -24.052-169.833  1.00 79.00           O
ATOM   6484  CG  LEU A 835     222.645 -23.303-165.688  1.00 79.00           C
ATOM   6485  CD1 LEU A 835     221.846 -24.010-164.594  1.00 79.00           C
ATOM   6486  CD2 LEU A 835     224.135 -23.453-165.359  1.00 79.00           C
ATOM   6487  N   GLU A 836     222.036 -24.403-170.146  1.00 77.50           N
ATOM   6488  CA  GLU A 836     222.170 -25.295-171.303  1.00 77.50           C
ATOM   6489  C   GLU A 836     222.909 -24.613-172.473  1.00 77.50           C
ATOM   6490  CB  GLU A 836     220.756 -25.792-171.654  1.00 77.50           C
ATOM   6491  O   GLU A 836     223.872 -25.168-173.009  1.00 77.50           O
ATOM   6492  CG  GLU A 836     220.697 -27.102-172.448  1.00 77.50           C
ATOM   6493  CD  GLU A 836     219.300 -27.738-172.310  1.00 77.50           C
ATOM   6494  OE1 GLU A 836     219.224 -28.903-171.855  1.00 77.50           O
ATOM   6495  OE2 GLU A 836     218.306 -27.041-172.623  1.00 77.50           O
***** protestar_10_10
ATOM   6474  N   ALA A 834     219.307 -21.054-169.741  1.00 77.18           N  <-- err: 0.0077
ATOM   6475  CA  ALA A 834     220.528 -20.251-169.741  1.00 77.18           C  <-- err: 0.0051
ATOM   6476  C   ALA A 834     221.782 -21.109-169.477  1.00 77.18           C  <-- err: 0.0057
ATOM   6477  CB  ALA A 834     220.374 -19.129-168.707  1.00 77.18           C  <-- err: 0.0033
ATOM   6478  O   ALA A 834     222.794 -20.966-170.170  1.00 77.18           O  <-- err: 0.0037
ATOM   6479  N   LEU A 835     221.705 -22.055-168.520  1.00 79.00           N  <-- err: 0.0042
ATOM   6480  CA  LEU A 835     222.761 -23.034-168.245  1.00 79.00           C  <-- err: 0.0032
ATOM   6481  C   LEU A 835     223.069 -23.892-169.466  1.00 79.00           C  <-- err: 0.0065
ATOM   6482  CB  LEU A 835     222.321 -23.914-167.057  1.00 79.00           C  <-- err: 0.0055
ATOM   6483  O   LEU A 835     224.235 -24.057-169.829  1.00 79.00           O  <-- err: 0.0071
ATOM   6484  CG  LEU A 835     222.640 -23.298-165.693  1.00 79.00           C  <-- err: 0.0087 ***
ATOM   6485  CD1 LEU A 835     221.848 -24.013-164.593  1.00 79.00           C  <-- err: 0.0037
ATOM   6486  CD2 LEU A 835     224.136 -23.452-165.363  1.00 79.00           C  <-- err: 0.0042
ATOM   6487  N   GLU A 836     222.035 -24.398-170.148  1.00 77.50           N  <-- err: 0.0055
ATOM   6488  CA  GLU A 836     222.167 -25.300-171.303  1.00 77.50           C  <-- err: 0.0058
ATOM   6489  C   GLU A 836     222.904 -24.618-172.469  1.00 77.50           C  <-- err: 0.0081
ATOM   6490  CB  GLU A 836     220.759 -25.795-171.655  1.00 77.50           C  <-- err: 0.0044
ATOM   6491  O   GLU A 836     223.872 -25.168-173.008  1.00 77.50           O  <-- err: 0.001
ATOM   6492  CG  GLU A 836     220.693 -27.104-172.447  1.00 77.50           C  <-- err: 0.0046
ATOM   6493  CD  GLU A 836     219.296 -27.742-172.315  1.00 77.50           C  <-- err: 0.0075
ATOM   6494  OE1 GLU A 836     219.219 -28.908-171.853  1.00 77.50           O  <-- err: 0.0073
ATOM   6495  OE2 GLU A 836     218.306 -27.038-172.623  1.00 77.50           O  <-- err: 0.003
```

```
*************** AF-Q6P5D4-F1-model_v4.pdb max_error: 0.0087
***** original
ATOM   9264  N   VAL A1132     299.283  -0.796-201.684  1.00 43.40           N
ATOM   9265  CA  VAL A1132     298.454   0.039-200.752  1.00 43.40           C
ATOM   9266  C   VAL A1132     296.977   0.470-201.056  1.00 43.40           C
ATOM   9267  CB  VAL A1132     299.321   1.241-200.281  1.00 43.40           C
ATOM   9268  O   VAL A1132     296.670   1.076-202.073  1.00 43.40           O
ATOM   9269  CG1 VAL A1132     298.667   2.177-199.261  1.00 43.40           C
ATOM   9270  CG2 VAL A1132     300.613   0.754-199.595  1.00 43.40           C
ATOM   9271  N   GLN A1133     296.134   0.315-200.008  1.00 52.17           N
ATOM   9272  CA  GLN A1133     294.946   1.107-199.566  1.00 52.17           C
ATOM   9273  C   GLN A1133     293.484   0.573-199.609  1.00 52.17           C
ATOM   9274  CB  GLN A1133     295.114   2.645-199.711  1.00 52.17           C
ATOM   9275  O   GLN A1133     293.229  -0.591-199.898  1.00 52.17           O
ATOM   9276  CG  GLN A1133     295.377   3.263-198.319  1.00 52.17           C
ATOM   9277  CD  GLN A1133     295.774   4.733-198.311  1.00 52.17           C
ATOM   9278  NE2 GLN A1133     295.652   5.396-197.182  1.00 52.17           N
ATOM   9279  OE1 GLN A1133     296.238   5.306-199.278  1.00 52.17           O
ATOM   9280  N   THR A1134     292.580   1.406-199.064  1.00 49.16           N
ATOM   9281  CA  THR A1134     291.345   1.154-198.265  1.00 49.16           C
ATOM   9282  C   THR A1134     290.255   2.206-198.653  1.00 49.16           C
ATOM   9283  CB  THR A1134     291.750   1.292-196.774  1.00 49.16           C
ATOM   9284  O   THR A1134     290.505   2.886-199.649  1.00 49.16           O
ATOM   9285  CG2 THR A1134     292.838   0.318-196.320  1.00 49.16           C
ATOM   9286  OG1 THR A1134     292.247   2.589-196.503  1.00 49.16           O
***** protestar_10_10
ATOM   9264  N   VAL A1132     299.288  -0.792-201.685  1.00 43.40           N  <-- err: 0.0065
ATOM   9265  CA  VAL A1132     298.452   0.044-200.750  1.00 43.40           C  <-- err: 0.0057
ATOM   9266  C   VAL A1132     296.978   0.473-201.058  1.00 43.40           C  <-- err: 0.0037
ATOM   9267  CB  VAL A1132     299.321   1.243-200.277  1.00 43.40           C  <-- err: 0.0045
ATOM   9268  O   VAL A1132     296.670   1.078-202.070  1.00 43.40           O  <-- err: 0.0036
ATOM   9269  CG1 VAL A1132     298.672   2.178-199.265  1.00 43.40           C  <-- err: 0.0065
ATOM   9270  CG2 VAL A1132     300.608   0.759-199.595  1.00 43.40           C  <-- err: 0.0071
ATOM   9271  N   GLN A1133     296.131   0.319-200.013  1.00 52.17           N  <-- err: 0.0071
ATOM   9272  CA  GLN A1133     294.943   1.111-199.562  1.00 52.17           C  <-- err: 0.0064
ATOM   9273  C   GLN A1133     293.480   0.572-199.606  1.00 52.17           C  <-- err: 0.0051
ATOM   9274  CB  GLN A1133     295.119   2.640-199.716  1.00 52.17           C  <-- err: 0.0087 ***
ATOM   9275  O   GLN A1133     293.227  -0.594-199.903  1.00 52.17           O  <-- err: 0.0062
ATOM   9276  CG  GLN A1133     295.372   3.267-198.319  1.00 52.17           C  <-- err: 0.0064
ATOM   9277  CD  GLN A1133     295.779   4.730-198.308  1.00 52.17           C  <-- err: 0.0066
ATOM   9278  NE2 GLN A1133     295.647   5.401-197.186  1.00 52.17           N  <-- err: 0.0081
ATOM   9279  OE1 GLN A1133     296.241   5.302-199.276  1.00 52.17           O  <-- err: 0.0054
ATOM   9280  N   THR A1134     292.578   1.408-199.067  1.00 49.16           N  <-- err: 0.0041
ATOM   9281  CA  THR A1134     291.346   1.155-198.264  1.00 49.16           C  <-- err: 0.0017
ATOM   9282  C   THR A1134     290.257   2.211-198.649  1.00 49.16           C  <-- err: 0.0067
ATOM   9283  CB  THR A1134     291.753   1.287-196.779  1.00 49.16           C  <-- err: 0.0077
ATOM   9284  O   THR A1134     290.510   2.882-199.650  1.00 49.16           O  <-- err: 0.0065
ATOM   9285  CG2 THR A1134     292.842   0.319-196.317  1.00 49.16           C  <-- err: 0.0051
ATOM   9286  OG1 THR A1134     292.248   2.585-196.504  1.00 49.16           O  <-- err: 0.0042
```

```
*************** AF-Q8CG73-F1-model_v4.pdb max_error: 0.0087
***** original
ATOM   1478  N   ALA A 186     143.611 -27.022 -72.572  1.00 25.64           N
ATOM   1479  CA  ALA A 186     144.831 -27.752 -72.086  1.00 25.64           C
ATOM   1480  C   ALA A 186     146.169 -26.897 -72.216  1.00 25.64           C
ATOM   1481  CB  ALA A 186     144.986 -29.097 -72.811  1.00 25.64           C
ATOM   1482  O   ALA A 186     146.140 -25.772 -72.698  1.00 25.64           O
ATOM   1483  N   GLU A 187     147.331 -27.424 -71.764  1.00 25.56           N
ATOM   1484  CA  GLU A 187     148.608 -26.816 -71.243  1.00 25.56           C
ATOM   1485  C   GLU A 187     149.780 -26.327 -72.198  1.00 25.56           C
ATOM   1486  CB  GLU A 187     149.222 -27.936 -70.364  1.00 25.56           C
ATOM   1487  O   GLU A 187     149.679 -26.408 -73.417  1.00 25.56           O
ATOM   1488  CG  GLU A 187     148.312 -28.581 -69.304  1.00 25.56           C
ATOM   1489  CD  GLU A 187     149.032 -29.722 -68.564  1.00 25.56           C
ATOM   1490  OE1 GLU A 187     148.880 -29.793 -67.325  1.00 25.56           O
ATOM   1491  OE2 GLU A 187     149.741 -30.504 -69.238  1.00 25.56           O
ATOM   1492  N   THR A 188     150.959 -25.956 -71.603  1.00 30.84           N
ATOM   1493  CA  THR A 188     152.398 -25.889 -72.103  1.00 30.84           C
ATOM   1494  C   THR A 188     153.051 -24.541 -72.578  1.00 30.84           C
ATOM   1495  CB  THR A 188     152.749 -27.010 -73.107  1.00 30.84           C
ATOM   1496  O   THR A 188     152.332 -23.620 -72.937  1.00 30.84           O
ATOM   1497  CG2 THR A 188     152.438 -28.420 -72.606  1.00 30.84           C
ATOM   1498  OG1 THR A 188     152.115 -26.825 -74.343  1.00 30.84           O
***** protestar_10_10
ATOM   1478  N   ALA A 186     143.616 -27.027 -72.567  1.00 25.64           N  <-- err: 0.0087 ***
ATOM   1479  CA  ALA A 186     144.826 -27.753 -72.083  1.00 25.64           C  <-- err: 0.0059
ATOM   1480  C   ALA A 186     146.168 -26.895 -72.215  1.00 25.64           C  <-- err: 0.0024
ATOM   1481  CB  ALA A 186     144.991 -29.095 -72.809  1.00 25.64           C  <-- err: 0.0057
ATOM   1482  O   ALA A 186     146.135 -25.773 -72.699  1.00 25.64           O  <-- err: 0.0052
ATOM   1483  N   GLU A 187     147.334 -27.423 -71.764  1.00 25.56           N  <-- err: 0.0032
ATOM   1484  CA  GLU A 187     148.610 -26.818 -71.247  1.00 25.56           C  <-- err: 0.0049
ATOM   1485  C   GLU A 187     149.776 -26.323 -72.193  1.00 25.56           C  <-- err: 0.0075
ATOM   1486  CB  GLU A 187     149.226 -27.940 -70.367  1.00 25.56           C  <-- err: 0.0064
ATOM   1487  O   GLU A 187     149.677 -26.411 -73.414  1.00 25.56           O  <-- err: 0.0047
ATOM   1488  CG  GLU A 187     148.313 -28.578 -69.300  1.00 25.56           C  <-- err: 0.0051
ATOM   1489  CD  GLU A 187     149.028 -29.722 -68.563  1.00 25.56           C  <-- err: 0.0041
ATOM   1490  OE1 GLU A 187     148.885 -29.788 -67.320  1.00 25.56           O  <-- err: 0.0087 ***
ATOM   1491  OE2 GLU A 187     149.743 -30.503 -69.234  1.00 25.56           O  <-- err: 0.0046
ATOM   1492  N   THR A 188     150.964 -25.960 -71.599  1.00 30.84           N  <-- err: 0.0075
ATOM   1493  CA  THR A 188     152.394 -25.894 -72.105  1.00 30.84           C  <-- err: 0.0067
ATOM   1494  C   THR A 188     153.054 -24.541 -72.578  1.00 30.84           C  <-- err: 0.003
ATOM   1495  CB  THR A 188     152.746 -27.005 -73.106  1.00 30.84           C  <-- err: 0.0059
ATOM   1496  O   THR A 188     152.328 -23.617 -72.941  1.00 30.84           O  <-- err: 0.0064
ATOM   1497  CG2 THR A 188     152.438 -28.424 -72.611  1.00 30.84           C  <-- err: 0.0064
ATOM   1498  OG1 THR A 188     152.119 -26.829 -74.338  1.00 30.84           O  <-- err: 0.0075
```

```
*************** AF-Q6NY15-F1-model_v4.pdb max_error: 0.0087
***** original
ATOM   3080  N   GLU A 381    -131.526 -52.475 118.983  1.00 74.18           N
ATOM   3081  CA  GLU A 381    -131.605 -53.022 117.612  1.00 74.18           C
ATOM   3082  C   GLU A 381    -131.172 -52.020 116.518  1.00 74.18           C
ATOM   3083  CB  GLU A 381    -133.033 -53.525 117.337  1.00 74.18           C
ATOM   3084  O   GLU A 381    -130.688 -52.414 115.456  1.00 74.18           O
ATOM   3085  CG  GLU A 381    -133.410 -54.747 118.195  1.00 74.18           C
ATOM   3086  CD  GLU A 381    -134.828 -55.282 117.918  1.00 74.18           C
ATOM   3087  OE1 GLU A 381    -135.157 -56.350 118.481  1.00 74.18           O
ATOM   3088  OE2 GLU A 381    -135.579 -54.629 117.155  1.00 74.18           O
ATOM   3089  N   LEU A 382    -131.279 -50.710 116.771  1.00 73.31           N
ATOM   3090  CA  LEU A 382    -130.898 -49.666 115.810  1.00 73.31           C
ATOM   3091  C   LEU A 382    -129.367 -49.502 115.673  1.00 73.31           C
ATOM   3092  CB  LEU A 382    -131.570 -48.348 116.242  1.00 73.31           C
ATOM   3093  O   LEU A 382    -128.884 -48.982 114.663  1.00 73.31           O
ATOM   3094  CG  LEU A 382    -131.737 -47.343 115.081  1.00 73.31           C
ATOM   3095  CD1 LEU A 382    -133.193 -47.283 114.614  1.00 73.31           C
ATOM   3096  CD2 LEU A 382    -131.311 -45.935 115.501  1.00 73.31           C
ATOM   3097  N   SER A 383    -128.598 -49.930 116.681  1.00 75.98           N
ATOM   3098  CA  SER A 383    -127.140 -49.756 116.732  1.00 75.98           C
ATOM   3099  C   SER A 383    -126.399 -50.717 115.786  1.00 75.98           C
ATOM   3100  CB  SER A 383    -126.667 -49.907 118.183  1.00 75.98           C
ATOM   3101  O   SER A 383    -125.472 -50.304 115.086  1.00 75.98           O
ATOM   3102  OG  SER A 383    -125.366 -49.376 118.336  1.00 75.98           O
***** protestar_10_10
ATOM   3080  N   GLU A 381    -131.527 -52.470 118.987  1.00 74.18           N  <-- err: 0.0065
ATOM   3081  CA  GLU A 381    -131.604 -53.020 117.612  1.00 74.18           C  <-- err: 0.0022
ATOM   3082  C   GLU A 381    -131.175 -52.019 116.523  1.00 74.18           C  <-- err: 0.0059
ATOM   3083  CB  GLU A 381    -133.034 -53.526 117.337  1.00 74.18           C  <-- err: 0.0014
ATOM   3084  O   GLU A 381    -130.691 -52.415 115.456  1.00 74.18           O  <-- err: 0.0032
ATOM   3085  CG  GLU A 381    -133.408 -54.747 118.195  1.00 74.18           C  <-- err: 0.002
ATOM   3086  CD  GLU A 381    -134.827 -55.286 117.920  1.00 74.18           C  <-- err: 0.0046
ATOM   3087  OE1 GLU A 381    -135.157 -56.353 118.481  1.00 74.18           O  <-- err: 0.003
ATOM   3088  OE2 GLU A 381    -135.575 -54.626 117.150  1.00 74.18           O  <-- err: 0.0071
ATOM   3089  N   LEU A 382    -131.274 -50.710 116.776  1.00 73.31           N  <-- err: 0.0071
ATOM   3090  CA  LEU A 382    -130.900 -49.665 115.808  1.00 73.31           C  <-- err: 0.003
ATOM   3091  C   LEU A 382    -129.371 -49.500 115.676  1.00 73.31           C  <-- err: 0.0054
ATOM   3092  CB  LEU A 382    -131.571 -48.345 116.237  1.00 73.31           C  <-- err: 0.0059
ATOM   3093  O   LEU A 382    -128.887 -48.983 114.664  1.00 73.31           O  <-- err: 0.0033
ATOM   3094  CG  LEU A 382    -131.736 -47.344 115.082  1.00 73.31           C  <-- err: 0.0017
ATOM   3095  CD1 LEU A 382    -133.188 -47.278 114.609  1.00 73.31           C  <-- err: 0.0087 ***
ATOM   3096  CD2 LEU A 382    -131.307 -45.936 115.500  1.00 73.31           C  <-- err: 0.0042
ATOM   3097  N   SER A 383    -128.601 -49.929 116.677  1.00 75.98           N  <-- err: 0.0051
ATOM   3098  CA  SER A 383    -127.138 -49.753 116.732  1.00 75.98           C  <-- err: 0.0036
ATOM   3099  C   SER A 383    -126.401 -50.721 115.786  1.00 75.98           C  <-- err: 0.0045
ATOM   3100  CB  SER A 383    -126.665 -49.907 118.184  1.00 75.98           C  <-- err: 0.0022
ATOM   3101  O   SER A 383    -125.477 -50.303 115.082  1.00 75.98           O  <-- err: 0.0065
ATOM   3102  OG  SER A 383    -125.367 -49.379 118.338  1.00 75.98           O  <-- err: 0.0037
```

5.4 ProteStAr 10/100

```
 *************** AF-A2A870-F1-model_v4.pdb max_error: 0.0987
***** original
ATOM   8199  N   GLN A1057    -150.700 -28.369 101.348  1.00 82.87           N
ATOM   8200  CA  GLN A1057    -151.505 -27.778 102.420  1.00 82.87           C
ATOM   8201  C   GLN A1057    -152.763 -27.095 101.869  1.00 82.87           C
ATOM   8202  CB  GLN A1057    -150.681 -26.802 103.275  1.00 82.87           C
ATOM   8203  O   GLN A1057    -153.830 -27.313 102.426  1.00 82.87           O
ATOM   8204  CG  GLN A1057    -149.761 -27.529 104.269  1.00 82.87           C
ATOM   8205  CD  GLN A1057    -148.973 -26.572 105.164  1.00 82.87           C
ATOM   8206  NE2 GLN A1057    -148.145 -27.081 106.049  1.00 82.87           N
ATOM   8207  OE1 GLN A1057    -149.076 -25.359 105.101  1.00 82.87           O
ATOM   8208  N   GLN A1058    -152.694 -26.343 100.764  1.00 83.62           N
ATOM   8209  CA  GLN A1058    -153.887 -25.777 100.116  1.00 83.62           C
ATOM   8210  C   GLN A1058    -154.826 -26.847  99.540  1.00 83.62           C
ATOM   8211  CB  GLN A1058    -153.510 -24.806  98.988  1.00 83.62           C
ATOM   8212  O   GLN A1058    -156.038 -26.668  99.580  1.00 83.62           O
ATOM   8213  CG  GLN A1058    -153.046 -23.434  99.494  1.00 83.62           C
ATOM   8214  CD  GLN A1058    -152.960 -22.395  98.376  1.00 83.62           C
ATOM   8215  NE2 GLN A1058    -152.470 -21.211  98.672  1.00 83.62           N
ATOM   8216  OE1 GLN A1058    -153.352 -22.597  97.233  1.00 83.62           O
ATOM   8217  N   GLU A1059    -154.306 -27.949  99.004  1.00 84.52           N
ATOM   8218  CA  GLU A1059    -155.111 -29.068  98.499  1.00 84.52           C
ATOM   8219  C   GLU A1059    -155.769 -29.850  99.637  1.00 84.52           C
ATOM   8220  CB  GLU A1059    -154.232 -30.005  97.664  1.00 84.52           C
ATOM   8221  O   GLU A1059    -156.949 -30.184  99.547  1.00 84.52           O
ATOM   8222  CG  GLU A1059    -153.870 -29.385  96.307  1.00 84.52           C
ATOM   8223  CD  GLU A1059    -152.868 -30.234  95.509  1.00 84.52           C
ATOM   8224  OE1 GLU A1059    -152.694 -29.909  94.314  1.00 84.52           O
ATOM   8225  OE2 GLU A1059    -152.283 -31.181  96.086  1.00 84.52           O
***** protestar_10_100
ATOM   8199  N   GLN A1057    -150.700 -28.369 101.343  1.00 82.87           N  <-- err: 0.005
ATOM   8200  CA  GLN A1057    -151.503 -27.775 102.421  1.00 82.87           C  <-- err: 0.0037
ATOM   8201  C   GLN A1057    -152.768 -27.093 101.871  1.00 82.87           C  <-- err: 0.0057
ATOM   8202  CB  GLN A1057    -150.650 -26.795 103.270  1.00 82.87           C  <-- err: 0.0322
ATOM   8203  O   GLN A1057    -153.870 -27.370 102.465  1.00 82.87           O  <-- err: 0.0798
ATOM   8204  CG  GLN A1057    -149.730 -27.485 104.305  1.00 82.87           C  <-- err: 0.0648
ATOM   8205  CD  GLN A1057    -148.925 -26.565 105.110  1.00 82.87           C  <-- err: 0.0726
ATOM   8206  NE2 GLN A1057    -148.120 -27.025 106.030  1.00 82.87           N  <-- err: 0.0642
ATOM   8207  OE1 GLN A1057    -149.040 -25.415 105.110  1.00 82.87           O  <-- err: 0.0672
ATOM   8208  N   GLN A1058    -152.691 -26.345 100.760  1.00 83.62           N  <-- err: 0.0054
ATOM   8209  CA  GLN A1058    -153.890 -25.773 100.111  1.00 83.62           C  <-- err: 0.0071
ATOM   8210  C   GLN A1058    -154.825 -26.851  99.539  1.00 83.62           C  <-- err: 0.0042
ATOM   8211  CB  GLN A1058    -153.525 -24.840  99.015  1.00 83.62           C  <-- err: 0.0459
ATOM   8212  O   GLN A1058    -156.055 -26.680  99.590  1.00 83.62           O  <-- err: 0.0231
ATOM   8213  CG  GLN A1058    -153.065 -23.460  99.475  1.00 83.62           C  <-- err: 0.0374
ATOM   8214  CD  GLN A1058    -152.950 -22.425  98.325  1.00 83.62           C  <-- err: 0.06
ATOM   8215  NE2 GLN A1058    -152.490 -21.160  98.670  1.00 83.62           N  <-- err: 0.0548
ATOM   8216  OE1 GLN A1058    -153.295 -22.540  97.290  1.00 83.62           O  <-- err: 0.0987 ***
ATOM   8217  N   GLU A1059    -154.308 -27.951  99.000  1.00 84.52           N  <-- err: 0.0049
ATOM   8218  CA  GLU A1059    -155.111 -29.073  98.494  1.00 84.52           C  <-- err: 0.0071
ATOM   8219  C   GLU A1059    -155.771 -29.854  99.638  1.00 84.52           C  <-- err: 0.0046
ATOM   8220  CB  GLU A1059    -154.215 -30.015  97.635  1.00 84.52           C  <-- err: 0.0351
ATOM   8221  O   GLU A1059    -156.975 -30.130  99.590  1.00 84.52           O  <-- err: 0.0738
ATOM   8222  CG  GLU A1059    -153.870 -29.440  96.255  1.00 84.52           C  <-- err: 0.0757
ATOM   8223  CD  GLU A1059    -152.835 -30.245  95.565  1.00 84.52           C  <-- err: 0.0659
ATOM   8224  OE1 GLU A1059    -152.720 -29.900  94.300  1.00 84.52           O  <-- err: 0.0309
ATOM   8225  OE2 GLU A1059    -152.260 -31.165  96.140  1.00 84.52           O  <-- err: 0.0608
```

```
 *************** AF-Q05722-F1-model_v4.pdb max_error: 0.0987
***** original
ATOM   3511  N   GLY A 478     -90.089  55.431  58.244  1.00 42.70           N
ATOM   3512  CA  GLY A 478     -91.202  55.062  59.104  1.00 42.70           C
ATOM   3513  C   GLY A 478     -92.489  55.420  58.378  1.00 42.70           C
ATOM   3514  O   GLY A 478     -92.738  56.601  58.191  1.00 42.70           O
ATOM   3515  N   ASP A 479     -93.194  54.404  57.869  1.00 44.23           N
ATOM   3516  CA  ASP A 479     -94.663  54.317  57.798  1.00 44.23           C
ATOM   3517  C   ASP A 479     -95.087  53.095  56.968  1.00 44.23           C
ATOM   3518  CB  ASP A 479     -95.352  55.594  57.266  1.00 44.23           C
ATOM   3519  O   ASP A 479     -95.204  53.135  55.741  1.00 44.23           O
ATOM   3520  CG  ASP A 479     -95.623  56.637  58.363  1.00 44.23           C
ATOM   3521  OD1 ASP A 479     -95.596  56.248  59.557  1.00 44.23           O
ATOM   3522  OD2 ASP A 479     -95.916  57.791  57.982  1.00 44.23           O
ATOM   3523  N   LYS A 480     -95.324  51.974  57.663  1.00 50.51           N
ATOM   3524  CA  LYS A 480     -96.167  50.862  57.187  1.00 50.51           C
ATOM   3525  C   LYS A 480     -96.690  50.043  58.375  1.00 50.51           C
ATOM   3526  CB  LYS A 480     -95.444  50.002  56.128  1.00 50.51           C
ATOM   3527  O   LYS A 480     -96.349  48.876  58.548  1.00 50.51           O
ATOM   3528  CG  LYS A 480     -96.506  49.230  55.330  1.00 50.51           C
ATOM   3529  CD  LYS A 480     -95.916  48.345  54.231  1.00 50.51           C
ATOM   3530  CE  LYS A 480     -97.095  47.682  53.508  1.00 50.51           C
ATOM   3531  NZ  LYS A 480     -96.652  46.717  52.474  1.00 50.51           N
***** protestar_10_100
ATOM   3511  N   GLY A 478     -90.090  55.429  58.245  1.00 42.70           N  <-- err: 0.0024
ATOM   3512  CA  GLY A 478     -91.201  55.066  59.103  1.00 42.70           C  <-- err: 0.0042
ATOM   3513  C   GLY A 478     -92.488  55.418  58.377  1.00 42.70           C  <-- err: 0.0024
ATOM   3514  O   GLY A 478     -92.690  56.580  58.190  1.00 42.70           O  <-- err: 0.0524
ATOM   3515  N   ASP A 479     -93.192  54.406  57.871  1.00 44.23           N  <-- err: 0.0035
ATOM   3516  CA  ASP A 479     -94.666  54.318  57.794  1.00 44.23           C  <-- err: 0.0051
ATOM   3517  C   ASP A 479     -95.084  53.097  56.969  1.00 44.23           C  <-- err: 0.0037
ATOM   3518  CB  ASP A 479     -95.335  55.545  57.270  1.00 44.23           C  <-- err: 0.052
ATOM   3519  O   ASP A 479     -95.220  53.130  55.775  1.00 44.23           O  <-- err: 0.0379
ATOM   3520  CG  ASP A 479     -95.680  56.580  58.420  1.00 44.23           C  <-- err: 0.0987 ***
ATOM   3521  OD1 ASP A 479     -95.565  56.235  59.570  1.00 44.23           O  <-- err: 0.036
ATOM   3522  OD2 ASP A 479     -95.910  57.845  57.960  1.00 44.23           O  <-- err: 0.0586
ATOM   3523  N   LYS A 480     -95.326  51.975  57.662  1.00 50.51           N  <-- err: 0.0024
ATOM   3524  CA  LYS A 480     -96.162  50.864  57.189  1.00 50.51           C  <-- err: 0.0057
ATOM   3525  C   LYS A 480     -96.690  50.039  58.377  1.00 50.51           C  <-- err: 0.0045
ATOM   3526  CB  LYS A 480     -95.450  50.025  56.120  1.00 50.51           C  <-- err: 0.0251
ATOM   3527  O   LYS A 480     -96.370  48.875  58.535  1.00 50.51           O  <-- err: 0.0247
ATOM   3528  CG  LYS A 480     -96.485  49.220  55.315  1.00 50.51           C  <-- err: 0.0277
ATOM   3529  CD  LYS A 480     -95.910  48.300  54.280  1.00 50.51           C  <-- err: 0.0668
ATOM   3530  CE  LYS A 480     -97.060  47.725  53.475  1.00 50.51           C  <-- err: 0.0645
ATOM   3531  NZ  LYS A 480     -96.600  46.690  52.440  1.00 50.51           N  <-- err: 0.0677
```

```
*************** AF-G3UXC7-F1-model_v4.pdb max_error: 0.0987
***** original
ATOM   3225  N   CYS A 413     -20.921 -39.239 -40.216  1.00 57.69           N
ATOM   3226  CA  CYS A 413     -19.951 -40.246 -39.785  1.00 57.69           C
ATOM   3227  C   CYS A 413     -18.786 -40.410 -40.778  1.00 57.69           C
ATOM   3228  CB  CYS A 413     -20.646 -41.604 -39.612  1.00 57.69           C
ATOM   3229  O   CYS A 413     -19.023 -40.835 -41.913  1.00 57.69           O
ATOM   3230  SG  CYS A 413     -21.778 -41.752 -38.210  1.00 57.69           S
ATOM   3231  N   PRO A 414     -17.525 -40.202 -40.351  1.00 55.29           N
ATOM   3232  CA  PRO A 414     -16.377 -40.814 -41.006  1.00 55.29           C
ATOM   3233  C   PRO A 414     -16.474 -42.341 -40.868  1.00 55.29           C
ATOM   3234  CB  PRO A 414     -15.130 -40.244 -40.313  1.00 55.29           C
ATOM   3235  O   PRO A 414     -16.984 -42.861 -39.875  1.00 55.29           O
ATOM   3236  CG  PRO A 414     -15.659 -39.062 -39.497  1.00 55.29           C
ATOM   3237  CD  PRO A 414     -17.078 -39.503 -39.158  1.00 55.29           C
ATOM   3238  N   SER A 415     -16.007 -43.075 -41.872  1.00 49.46           N
ATOM   3239  CA  SER A 415     -16.136 -44.534 -41.936  1.00 49.46           C
ATOM   3240  C   SER A 415     -15.154 -45.271 -41.011  1.00 49.46           C
ATOM   3241  CB  SER A 415     -15.911 -44.976 -43.388  1.00 49.46           C
ATOM   3242  O   SER A 415     -13.949 -45.175 -41.247  1.00 49.46           O
ATOM   3243  OG  SER A 415     -14.711 -44.408 -43.886  1.00 49.46           O
***** protestar_10_100
ATOM   3225  N   CYS A 413     -20.922 -39.237 -40.216  1.00 57.69           N  <-- err: 0.0022
ATOM   3226  CA  CYS A 413     -19.954 -40.249 -39.787  1.00 57.69           C  <-- err: 0.0047
ATOM   3227  C   CYS A 413     -18.788 -40.414 -40.777  1.00 57.69           C  <-- err: 0.0046
ATOM   3228  CB  CYS A 413     -20.700 -41.630 -39.560  1.00 57.69           C  <-- err: 0.0793
ATOM   3229  O   CYS A 413     -18.975 -40.825 -41.860  1.00 57.69           O  <-- err: 0.0722
ATOM   3230  SG  CYS A 413     -21.735 -41.745 -38.180  1.00 57.69           S  <-- err: 0.0529
ATOM   3231  N   PRO A 414     -17.523 -40.205 -40.348  1.00 55.29           N  <-- err: 0.0047
ATOM   3232  CA  PRO A 414     -16.379 -40.810 -41.008  1.00 55.29           C  <-- err: 0.0049
ATOM   3233  C   PRO A 414     -16.478 -42.339 -40.865  1.00 55.29           C  <-- err: 0.0054
ATOM   3234  CB  PRO A 414     -15.180 -40.250 -40.365  1.00 55.29           C  <-- err: 0.0724
ATOM   3235  O   PRO A 414     -17.020 -42.895 -39.905  1.00 55.29           O  <-- err: 0.0579
ATOM   3236  CG  PRO A 414     -15.640 -39.100 -39.445  1.00 55.29           C  <-- err: 0.0671
ATOM   3237  CD  PRO A 414     -17.135 -39.560 -39.215  1.00 55.29           C  <-- err: 0.0987 ***
ATOM   3238  N   SER A 415     -16.005 -43.076 -41.877  1.00 49.46           N  <-- err: 0.0055
ATOM   3239  CA  SER A 415     -16.137 -44.539 -41.932  1.00 49.46           C  <-- err: 0.0065
ATOM   3240  C   SER A 415     -15.158 -45.276 -41.008  1.00 49.46           C  <-- err: 0.0071
ATOM   3241  CB  SER A 415     -15.870 -44.965 -43.355  1.00 49.46           C  <-- err: 0.0538
ATOM   3242  O   SER A 415     -13.915 -45.195 -41.285  1.00 49.46           O  <-- err: 0.0548
ATOM   3243  OG  SER A 415     -14.720 -44.390 -43.930  1.00 49.46           O  <-- err: 0.0484
```

```
*************** AF-E9PUT5-F1-model_v4.pdb max_error: 0.0987
***** original
ATOM   6619  N   LEU A 824     -59.131 -21.802  56.989  1.00 91.60           N
ATOM   6620  CA  LEU A 824     -60.228 -21.215  57.765  1.00 91.60           C
ATOM   6621  C   LEU A 824     -61.567 -21.898  57.451  1.00 91.60           C
ATOM   6622  CB  LEU A 824     -60.298 -19.699  57.499  1.00 91.60           C
ATOM   6623  O   LEU A 824     -62.299 -22.259  58.375  1.00 91.60           O
ATOM   6624  CG  LEU A 824     -59.165 -18.879  58.147  1.00 91.60           C
ATOM   6625  CD1 LEU A 824     -59.228 -17.436  57.645  1.00 91.60           C
ATOM   6626  CD2 LEU A 824     -59.280 -18.849  59.674  1.00 91.60           C
ATOM   6627  N   LEU A 825     -61.876 -22.138  56.172  1.00 93.57           N
ATOM   6628  CA  LEU A 825     -63.101 -22.827  55.760  1.00 93.57           C
ATOM   6629  C   LEU A 825     -63.165 -24.259  56.316  1.00 93.57           C
ATOM   6630  CB  LEU A 825     -63.192 -22.828  54.223  1.00 93.57           C
ATOM   6631  O   LEU A 825     -64.123 -24.632  56.998  1.00 93.57           O
ATOM   6632  CG  LEU A 825     -64.502 -23.437  53.683  1.00 93.57           C
ATOM   6633  CD1 LEU A 825     -65.696 -22.525  53.972  1.00 93.57           C
ATOM   6634  CD2 LEU A 825     -64.387 -23.669  52.181  1.00 93.57           C
ATOM   6635  N   GLY A 826     -62.138 -25.061  56.037  1.00 89.86           N
ATOM   6636  CA  GLY A 826     -62.092 -26.482  56.362  1.00 89.86           C
ATOM   6637  C   GLY A 826     -62.094 -26.745  57.864  1.00 89.86           C
ATOM   6638  O   GLY A 826     -62.851 -27.592  58.335  1.00 89.86           O
***** protestar_10_100
ATOM   6619  N   LEU A 824     -59.136 -21.802  56.991  1.00 91.60           N  <-- err: 0.0054
ATOM   6620  CA  LEU A 824     -60.225 -21.219  57.761  1.00 91.60           C  <-- err: 0.0064
ATOM   6621  C   LEU A 824     -61.567 -21.901  57.453  1.00 91.60           C  <-- err: 0.0036
ATOM   6622  CB  LEU A 824     -60.260 -19.665  57.500  1.00 91.60           C  <-- err: 0.051
ATOM   6623  O   LEU A 824     -62.330 -22.310  58.420  1.00 91.60           O  <-- err: 0.0747
ATOM   6624  CG  LEU A 824     -59.110 -18.860  58.190  1.00 91.60           C  <-- err: 0.0724
ATOM   6625  CD1 LEU A 824     -59.225 -17.480  57.615  1.00 91.60           C  <-- err: 0.0533
ATOM   6626  CD2 LEU A 824     -59.225 -18.860  59.685  1.00 91.60           C  <-- err: 0.0572
ATOM   6627  N   LEU A 825     -61.875 -22.143  56.177  1.00 93.57           N  <-- err: 0.0071
ATOM   6628  CA  LEU A 825     -63.096 -22.825  55.759  1.00 93.57           C  <-- err: 0.0055
ATOM   6629  C   LEU A 825     -63.162 -24.255  56.320  1.00 93.57           C  <-- err: 0.0064
ATOM   6630  CB  LEU A 825     -63.135 -22.885  54.280  1.00 93.57           C  <-- err: 0.0987 ***
ATOM   6631  O   LEU A 825     -64.170 -24.610  57.040  1.00 93.57           O  <-- err: 0.0668
ATOM   6632  CG  LEU A 825     -64.515 -23.460  53.705  1.00 93.57           C  <-- err: 0.0344
ATOM   6633  CD1 LEU A 825     -65.665 -22.540  53.935  1.00 93.57           C  <-- err: 0.0505
ATOM   6634  CD2 LEU A 825     -64.400 -23.690  52.210  1.00 93.57           C  <-- err: 0.0381
ATOM   6635  N   GLY A 826     -62.139 -25.058  56.034  1.00 89.86           N  <-- err: 0.0044
ATOM   6636  CA  GLY A 826     -62.095 -26.477  56.364  1.00 89.86           C  <-- err: 0.0062
ATOM   6637  C   GLY A 826     -62.095 -26.741  57.860  1.00 89.86           C  <-- err: 0.0057
ATOM   6638  O   GLY A 826     -62.905 -27.600  58.305  1.00 89.86           O  <-- err: 0.0623
```

```
*************** AF-A0A1D5RMD1-F1-model_v4.pdb max_error: 0.0987
***** original
ATOM   6057  N   GLU A 804     -31.360  44.111  22.438  1.00 35.14           N
ATOM   6058  CA  GLU A 804     -30.906  43.464  23.703  1.00 35.14           C
ATOM   6059  C   GLU A 804     -30.430  41.977  23.488  1.00 35.14           C
ATOM   6060  CB  GLU A 804     -32.064  43.696  24.703  1.00 35.14           C
ATOM   6061  O   GLU A 804     -29.972  41.698  22.383  1.00 35.14           O
ATOM   6062  CG  GLU A 804     -31.812  44.812  25.730  1.00 35.14           C
ATOM   6063  CD  GLU A 804     -32.195  44.317  27.129  1.00 35.14           C
ATOM   6064  OE1 GLU A 804     -31.378  43.555  27.699  1.00 35.14           O
ATOM   6065  OE2 GLU A 804     -33.322  44.618  27.570  1.00 35.14           O
ATOM   6066  N   THR A 805     -30.364  40.973  24.400  1.00 35.29           N
ATOM   6067  CA  THR A 805     -30.909  40.737  25.770  1.00 35.29           C
ATOM   6068  C   THR A 805     -30.092  39.701  26.599  1.00 35.29           C
ATOM   6069  CB  THR A 805     -32.372  40.192  25.702  1.00 35.29           C
ATOM   6070  O   THR A 805     -29.160  39.070  26.106  1.00 35.29           O
ATOM   6071  CG2 THR A 805     -33.260  40.617  26.881  1.00 35.29           C
ATOM   6072  OG1 THR A 805     -33.084  40.601  24.555  1.00 35.29           O
ATOM   6073  N   ALA A 806     -30.486  39.532  27.871  1.00 37.59           N
ATOM   6074  CA  ALA A 806     -30.176  38.513  28.900  1.00 37.59           C
ATOM   6075  C   ALA A 806     -29.980  37.033  28.474  1.00 37.59           C
ATOM   6076  CB  ALA A 806     -31.423  38.565  29.806  1.00 37.59           C
ATOM   6077  O   ALA A 806     -30.573  36.597  27.493  1.00 37.59           O
***** protestar_10_100
ATOM   6057  N   GLU A 804     -31.361  44.110  22.440  1.00 35.14           N  <-- err: 0.0024
ATOM   6058  CA  GLU A 804     -30.910  43.461  23.705  1.00 35.14           C  <-- err: 0.0054
ATOM   6059  C   GLU A 804     -30.426  41.976  23.485  1.00 35.14           C  <-- err: 0.0051
ATOM   6060  CB  GLU A 804     -32.085  43.700  24.725  1.00 35.14           C  <-- err: 0.0307
ATOM   6061  O   GLU A 804     -30.015  41.745  22.425  1.00 35.14           O  <-- err: 0.0763
ATOM   6062  CG  GLU A 804     -31.855  44.850  25.760  1.00 35.14           C  <-- err: 0.0648
ATOM   6063  CD  GLU A 804     -32.200  44.275  27.140  1.00 35.14           C  <-- err: 0.0437
ATOM   6064  OE1 GLU A 804     -31.395  43.585  27.715  1.00 35.14           O  <-- err: 0.038
ATOM   6065  OE2 GLU A 804     -33.350  44.620  27.600  1.00 35.14           O  <-- err: 0.0411
ATOM   6066  N   THR A 805     -30.360  40.975  24.398  1.00 35.29           N  <-- err: 0.0049
ATOM   6067  CA  THR A 805     -30.910  40.733  25.773  1.00 35.29           C  <-- err: 0.0051
ATOM   6068  C   THR A 805     -30.096  39.699  26.598  1.00 35.29           C  <-- err: 0.0046
ATOM   6069  CB  THR A 805     -32.315  40.135  25.645  1.00 35.29           C  <-- err: 0.0987 ***
ATOM   6070  O   THR A 805     -29.210  39.100  26.105  1.00 35.29           O  <-- err: 0.0583
ATOM   6071  CG2 THR A 805     -33.235  40.595  26.910  1.00 35.29           C  <-- err: 0.0442
ATOM   6072  OG1 THR A 805     -33.120  40.595  24.610  1.00 35.29           O  <-- err: 0.066
ATOM   6073  N   ALA A 806     -30.481  39.534  27.874  1.00 37.59           N  <-- err: 0.0062
ATOM   6074  CA  ALA A 806     -30.173  38.511  28.897  1.00 37.59           C  <-- err: 0.0047
ATOM   6075  C   ALA A 806     -29.975  37.037  28.479  1.00 37.59           C  <-- err: 0.0081
ATOM   6076  CB  ALA A 806     -31.395  38.525  29.785  1.00 37.59           C  <-- err: 0.0532
ATOM   6077  O   ALA A 806     -30.590  36.570  27.485  1.00 37.59           O  <-- err: 0.0329
```

5.5 ProteStAr 80/140

```
*************** AF-Q80TK0-F1-model_v4.pdb max_error: 0.1386
***** original
ATOM   4241  N   ALA A 570     -28.495 -37.215  61.881  1.00 35.22           N
ATOM   4242  CA  ALA A 570     -28.335 -38.485  61.151  1.00 35.22           C
ATOM   4243  C   ALA A 570     -29.695 -39.114  60.865  1.00 35.22           C
ATOM   4244  CB  ALA A 570     -27.466 -39.469  61.957  1.00 35.22           C
ATOM   4245  O   ALA A 570     -30.553 -39.197  61.747  1.00 35.22           O
ATOM   4246  N   TYR A 571     -30.042 -39.231  59.529  1.00 33.54           N
ATOM   4247  CA  TYR A 571     -30.123 -40.431  58.680  1.00 33.54           C
ATOM   4248  C   TYR A 571     -31.578 -40.817  58.442  1.00 33.54           C
ATOM   4249  CB  TYR A 571     -29.382 -41.618  59.328  1.00 33.54           C
ATOM   4250  O   TYR A 571     -32.385 -40.850  59.374  1.00 33.54           O
ATOM   4251  CG  TYR A 571     -27.960 -41.767  58.819  1.00 33.54           C
ATOM   4252  CD1 TYR A 571     -27.696 -42.513  57.670  1.00 33.54           C
ATOM   4253  CD2 TYR A 571     -26.890 -41.171  59.492  1.00 33.54           C
ATOM   4254  CE1 TYR A 571     -26.384 -42.664  57.195  1.00 33.54           C
ATOM   4255  CE2 TYR A 571     -25.578 -41.324  59.038  1.00 33.54           C
ATOM   4256  OH  TYR A 571     -24.026 -42.208  57.429  1.00 33.54           O
ATOM   4257  CZ  TYR A 571     -25.345 -42.064  57.890  1.00 33.54           C
ATOM   4258  N   LEU A 572     -32.018 -40.802  57.051  1.00 35.30           N
ATOM   4259  CA  LEU A 572     -32.402 -41.824  56.062  1.00 35.30           C
ATOM   4260  C   LEU A 572     -33.652 -42.572  56.511  1.00 35.30           C
ATOM   4261  CB  LEU A 572     -31.257 -42.830  55.842  1.00 35.30           C
ATOM   4262  O   LEU A 572     -33.735 -43.035  57.651  1.00 35.30           O
ATOM   4263  CG  LEU A 572     -30.159 -42.373  54.864  1.00 35.30           C
ATOM   4264  CD1 LEU A 572     -28.977 -43.358  54.911  1.00 35.30           C
ATOM   4265  CD2 LEU A 572     -30.717 -42.297  53.437  1.00 35.30           C
***** protestar_80_140
ATOM   4241  N   ALA A 570     -28.520 -37.260  61.916  1.00 35.22           N  <-- err: 0.0622
ATOM   4242  CA  ALA A 570     -28.336 -38.456  61.180  1.00 35.22           C  <-- err: 0.041
ATOM   4243  C   ALA A 570     -29.716 -39.100  60.904  1.00 35.22           C  <-- err: 0.0465
ATOM   4244  CB  ALA A 570     -27.531 -39.445  61.985  1.00 35.22           C  <-- err: 0.0747
ATOM   4245  O   ALA A 570     -30.590 -39.123  61.824  1.00 35.22           O  <-- err: 0.113
ATOM   4246  N   TYR A 571     -30.084 -39.192  59.524  1.00 33.54           N  <-- err: 0.0575
ATOM   4247  CA  TYR A 571     -30.084 -40.388  58.696  1.00 33.54           C  <-- err: 0.0602
ATOM   4248  C   TYR A 571     -31.556 -40.848  58.420  1.00 33.54           C  <-- err: 0.0439
ATOM   4249  CB  TYR A 571     -29.302 -41.538  59.248  1.00 33.54           C  <-- err: 0.1386 ***
ATOM   4250  O   TYR A 571     -32.361 -40.894  59.409  1.00 33.54           O  <-- err: 0.0611
ATOM   4251  CG  TYR A 571     -28.014 -41.699  58.765  1.00 33.54           C  <-- err: 0.1023
ATOM   4252  CD1 TYR A 571     -27.692 -42.504  57.638  1.00 33.54           C  <-- err: 0.0335
ATOM   4253  CD2 TYR A 571     -26.887 -41.216  59.570  1.00 33.54           C  <-- err: 0.0901
ATOM   4254  CE1 TYR A 571     -26.404 -42.665  57.155  1.00 33.54           C  <-- err: 0.0447
ATOM   4255  CE2 TYR A 571     -25.599 -41.377  59.087  1.00 33.54           C  <-- err: 0.0752
ATOM   4256  OH  TYR A 571     -23.989 -42.182  57.477  1.00 33.54           O  <-- err: 0.0659
ATOM   4257  CZ  TYR A 571     -25.277 -42.021  57.960  1.00 33.54           C  <-- err: 0.1066
ATOM   4258  N   LEU A 572     -32.016 -40.848  57.040  1.00 35.30           N  <-- err: 0.0473
ATOM   4259  CA  LEU A 572     -32.384 -41.860  56.028  1.00 35.30           C  <-- err: 0.0527
ATOM   4260  C   LEU A 572     -33.672 -42.596  56.488  1.00 35.30           C  <-- err: 0.0388
ATOM   4261  CB  LEU A 572     -31.234 -42.826  55.867  1.00 35.30           C  <-- err: 0.0342
ATOM   4262  O   LEU A 572     -33.810 -42.987  57.638  1.00 35.30           O  <-- err: 0.09
ATOM   4263  CG  LEU A 572     -30.107 -42.343  54.901  1.00 35.30           C  <-- err: 0.0705
ATOM   4264  CD1 LEU A 572     -28.980 -43.309  54.901  1.00 35.30           C  <-- err: 0.0501
ATOM   4265  CD2 LEU A 572     -30.751 -42.343  53.452  1.00 35.30           C  <-- err: 0.0591
```

```
*************** AF-Q9WVH6-F1-model_v4.pdb max_error: 0.1386
***** original
ATOM    879  N   GLU A 114     122.961   2.157 -75.849  1.00 91.53           N
ATOM    880  CA  GLU A 114     122.921   2.756 -74.514  1.00 91.53           C
ATOM    881  C   GLU A 114     121.698   3.672 -74.350  1.00 91.53           C
ATOM    882  CB  GLU A 114     124.219   3.547 -74.288  1.00 91.53           C
ATOM    883  O   GLU A 114     120.956   3.550 -73.373  1.00 91.53           O
ATOM    884  CG  GLU A 114     124.347   4.045 -72.841  1.00 91.53           C
ATOM    885  CD  GLU A 114     125.440   5.103 -72.647  1.00 91.53           C
ATOM    886  OE1 GLU A 114     125.439   5.686 -71.537  1.00 91.53           O
ATOM    887  OE2 GLU A 114     126.185   5.418 -73.595  1.00 91.53           O
ATOM    888  N   GLN A 115     121.440   4.547 -75.328  1.00 91.65           N
ATOM    889  CA  GLN A 115     120.275   5.431 -75.330  1.00 91.65           C
ATOM    890  C   GLN A 115     118.967   4.626 -75.330  1.00 91.65           C
ATOM    891  CB  GLN A 115     120.347   6.360 -76.555  1.00 91.65           C
ATOM    892  O   GLN A 115     118.044   4.944 -74.580  1.00 91.65           O
ATOM    893  CG  GLN A 115     119.442   7.596 -76.440  1.00 91.65           C
ATOM    894  CD  GLN A 115     120.017   8.695 -75.548  1.00 91.65           C
ATOM    895  NE2 GLN A 115     119.352   9.824 -75.443  1.00 91.65           N
ATOM    896  OE1 GLN A 115     121.056   8.585 -74.923  1.00 91.65           O
ATOM    897  N   SER A 116     118.890   3.566 -76.136  1.00 92.18           N
ATOM    898  CA  SER A 116     117.711   2.704 -76.236  1.00 92.18           C
ATOM    899  C   SER A 116     117.463   1.931 -74.938  1.00 92.18           C
ATOM    900  CB  SER A 116     117.863   1.762 -77.432  1.00 92.18           C
ATOM    901  O   SER A 116     116.346   1.964 -74.424  1.00 92.18           O
ATOM    902  OG  SER A 116     116.607   1.233 -77.804  1.00 92.18           O
***** protestar_80_140
ATOM    879  N   GLU A 114     123.004   2.116 -75.808  1.00 91.53           N  <-- err: 0.0722
ATOM    880  CA  GLU A 114     122.912   2.760 -74.520  1.00 91.53           C  <-- err: 0.0115
ATOM    881  C   GLU A 114     121.716   3.680 -74.336  1.00 91.53           C  <-- err: 0.0242
ATOM    882  CB  GLU A 114     124.292   3.542 -74.221  1.00 91.53           C  <-- err: 0.0992
ATOM    883  O   GLU A 114     120.911   3.542 -73.416  1.00 91.53           O  <-- err: 0.0628
ATOM    884  CG  GLU A 114     124.292   4.025 -72.772  1.00 91.53           C  <-- err: 0.0905
ATOM    885  CD  GLU A 114     125.419   5.152 -72.611  1.00 91.53           C  <-- err: 0.0643
ATOM    886  OE1 GLU A 114     125.419   5.635 -71.484  1.00 91.53           O  <-- err: 0.0762
ATOM    887  OE2 GLU A 114     126.224   5.474 -73.577  1.00 91.53           O  <-- err: 0.0706
ATOM    888  N   GLN A 115     121.440   4.508 -75.348  1.00 91.65           N  <-- err: 0.0438
ATOM    889  CA  GLN A 115     120.244   5.428 -75.348  1.00 91.65           C  <-- err: 0.036
ATOM    890  C   GLN A 115     118.956   4.600 -75.348  1.00 91.65           C  <-- err: 0.0335
ATOM    891  CB  GLN A 115     120.267   6.440 -76.475  1.00 91.65           C  <-- err: 0.1386 ***
ATOM    892  O   GLN A 115     118.013   4.991 -74.543  1.00 91.65           O  <-- err: 0.0674
ATOM    893  CG  GLN A 115     119.462   7.567 -76.475  1.00 91.65           C  <-- err: 0.0497
ATOM    894  CD  GLN A 115     119.945   8.694 -75.509  1.00 91.65           C  <-- err: 0.0819
ATOM    895  NE2 GLN A 115     119.301   9.821 -75.509  1.00 91.65           N  <-- err: 0.0835
ATOM    896  OE1 GLN A 115     121.072   8.533 -74.865  1.00 91.65           O  <-- err: 0.0795
ATOM    897  N   SER A 116     118.864   3.588 -76.176  1.00 92.18           N  <-- err: 0.0525
ATOM    898  CA  SER A 116     117.668   2.668 -76.268  1.00 92.18           C  <-- err: 0.0646
ATOM    899  C   SER A 116     117.484   1.932 -74.980  1.00 92.18           C  <-- err: 0.047
ATOM    900  CB  SER A 116     117.852   1.771 -77.441  1.00 92.18           C  <-- err: 0.0168
ATOM    901  O   SER A 116     116.403   1.932 -74.382  1.00 92.18           O  <-- err: 0.0777
ATOM    902  OG  SER A 116     116.564   1.288 -77.763  1.00 92.18           O  <-- err: 0.081
```

```
*************** AF-Q3TYU2-F1-model_v4.pdb max_error: 0.1386
***** original
ATOM   4597  N   GLY A 582      46.370  17.884  -8.492  1.00 77.45           N
ATOM   4598  CA  GLY A 582      46.340  18.295  -7.087  1.00 77.45           C
ATOM   4599  C   GLY A 582      47.643  17.960  -6.343  1.00 77.45           C
ATOM   4600  O   GLY A 582      48.531  17.312  -6.894  1.00 77.45           O
ATOM   4601  N   PRO A 583      47.773  18.338  -5.056  1.00 74.91           N
ATOM   4602  CA  PRO A 583      49.002  18.135  -4.276  1.00 74.91           C
ATOM   4603  C   PRO A 583      49.320  16.662  -3.974  1.00 74.91           C
ATOM   4604  CB  PRO A 583      48.774  18.924  -2.981  1.00 74.91           C
ATOM   4605  O   PRO A 583      50.452  16.339  -3.630  1.00 74.91           O
ATOM   4606  CG  PRO A 583      47.254  18.918  -2.818  1.00 74.91           C
ATOM   4607  CD  PRO A 583      46.755  19.001  -4.259  1.00 74.91           C
ATOM   4608  N   LYS A 584      48.321  15.774  -4.066  1.00 73.52           N
ATOM   4609  CA  LYS A 584      48.458  14.322  -3.859  1.00 73.52           C
ATOM   4610  C   LYS A 584      48.614  13.543  -5.171  1.00 73.52           C
ATOM   4611  CB  LYS A 584      47.256  13.787  -3.066  1.00 73.52           C
ATOM   4612  O   LYS A 584      48.718  12.321  -5.129  1.00 73.52           O
ATOM   4613  CG  LYS A 584      47.169  14.335  -1.635  1.00 73.52           C
ATOM   4614  CD  LYS A 584      45.962  13.705  -0.932  1.00 73.52           C
ATOM   4615  CE  LYS A 584      45.833  14.209   0.506  1.00 73.52           C
ATOM   4616  NZ  LYS A 584      44.638  13.619   1.159  1.00 73.52           N
***** protestar_80_140
ATOM   4597  N   GLY A 582      46.368  17.848  -8.464  1.00 77.45           N  <-- err: 0.0457
ATOM   4598  CA  GLY A 582      46.368  18.308  -7.084  1.00 77.45           C  <-- err: 0.031
ATOM   4599  C   GLY A 582      47.656  17.940  -6.348  1.00 77.45           C  <-- err: 0.0244
ATOM   4600  O   GLY A 582      48.461  17.388  -6.923  1.00 77.45           O  <-- err: 0.1073
ATOM   4601  N   PRO A 583      47.748  18.308  -5.060  1.00 74.91           N  <-- err: 0.0393
ATOM   4602  CA  PRO A 583      49.036  18.124  -4.232  1.00 74.91           C  <-- err: 0.0567
ATOM   4603  C   PRO A 583      49.312  16.652  -3.956  1.00 74.91           C  <-- err: 0.0221
ATOM   4604  CB  PRO A 583      48.783  18.998  -3.059  1.00 74.91           C  <-- err: 0.1079
ATOM   4605  O   PRO A 583      50.393  16.261  -3.703  1.00 74.91           O  <-- err: 0.122
ATOM   4606  CG  PRO A 583      47.334  18.998  -2.898  1.00 74.91           C  <-- err: 0.1386 ***
ATOM   4607  CD  PRO A 583      46.690  18.998  -4.186  1.00 74.91           C  <-- err: 0.0978
ATOM   4608  N   LYS A 584      48.300  15.732  -4.048  1.00 73.52           N  <-- err: 0.0503
ATOM   4609  CA  LYS A 584      48.484  14.352  -3.864  1.00 73.52           C  <-- err: 0.04
ATOM   4610  C   LYS A 584      48.576  13.524  -5.152  1.00 73.52           C  <-- err: 0.0465
ATOM   4611  CB  LYS A 584      47.334  13.846  -3.059  1.00 73.52           C  <-- err: 0.0981
ATOM   4612  O   LYS A 584      48.783  12.397  -5.152  1.00 73.52           O  <-- err: 0.1026
ATOM   4613  CG  LYS A 584      47.173  14.329  -1.610  1.00 73.52           C  <-- err: 0.026
ATOM   4614  CD  LYS A 584      45.885  13.685  -0.966  1.00 73.52           C  <-- err: 0.0865
ATOM   4615  CE  LYS A 584      45.885  14.168   0.483  1.00 73.52           C  <-- err: 0.0701
ATOM   4616  NZ  LYS A 584      44.597  13.685   1.127  1.00 73.52           N  <-- err: 0.084
```

```
*************** AF-P27792-F1-model_v4.pdb max_error: 0.1386
***** original
ATOM   2104  N   VAL A 277       9.286  55.605 -28.616  1.00 64.82           N
ATOM   2105  CA  VAL A 277       9.403  57.023 -28.984  1.00 64.82           C
ATOM   2106  C   VAL A 277       9.033  57.148 -30.461  1.00 64.82           C
ATOM   2107  CB  VAL A 277      10.813  57.594 -28.699  1.00 64.82           C
ATOM   2108  O   VAL A 277       9.736  56.604 -31.304  1.00 64.82           O
ATOM   2109  CG1 VAL A 277      11.049  58.975 -29.333  1.00 64.82           C
ATOM   2110  CG2 VAL A 277      11.032  57.753 -27.187  1.00 64.82           C
ATOM   2111  N   ARG A 278       7.903  57.832 -30.676  1.00 49.84           N
ATOM   2112  CA  ARG A 278       7.540  58.808 -31.722  1.00 49.84           C
ATOM   2113  C   ARG A 278       8.039  58.632 -33.158  1.00 49.84           C
ATOM   2114  CB  ARG A 278       7.874  60.214 -31.201  1.00 49.84           C
ATOM   2115  O   ARG A 278       9.266  58.657 -33.374  1.00 49.84           O
ATOM   2116  CG  ARG A 278       6.813  61.234 -31.622  1.00 49.84           C
ATOM   2117  CD  ARG A 278       7.200  62.609 -31.069  1.00 49.84           C
ATOM   2118  NE  ARG A 278       6.038  63.514 -30.993  1.00 49.84           N
ATOM   2119  NH1 ARG A 278       6.393  64.326 -28.871  1.00 49.84           N
ATOM   2120  NH2 ARG A 278       4.617  65.006 -30.031  1.00 49.84           N
ATOM   2121  CZ  ARG A 278       5.692  64.274 -29.970  1.00 49.84           C
ATOM   2122  OXT ARG A 278       7.133  58.700 -34.014  1.00 49.84           O
***** protestar_80_140
ATOM   2104  N   VAL A 277       9.292  55.568 -28.612  1.00 64.82           N  <-- err: 0.0377
ATOM   2105  CA  VAL A 277       9.384  57.040 -28.980  1.00 64.82           C  <-- err: 0.0258
ATOM   2106  C   VAL A 277       9.016  57.132 -30.452  1.00 64.82           C  <-- err: 0.025
ATOM   2107  CB  VAL A 277      10.787  57.638 -28.658  1.00 64.82           C  <-- err: 0.0655
ATOM   2108  O   VAL A 277       9.660  56.672 -31.234  1.00 64.82           O  <-- err: 0.1237
ATOM   2109  CG1 VAL A 277      11.109  58.926 -29.302  1.00 64.82           C  <-- err: 0.0834
ATOM   2110  CG2 VAL A 277      11.109  57.799 -27.209  1.00 64.82           C  <-- err: 0.0924
ATOM   2111  N   ARG A 278       7.912  57.868 -30.636  1.00 49.84           N  <-- err: 0.0546
ATOM   2112  CA  ARG A 278       7.544  58.788 -31.740  1.00 49.84           C  <-- err: 0.0272
ATOM   2113  C   ARG A 278       8.004  58.604 -33.120  1.00 49.84           C  <-- err: 0.0588
ATOM   2114  CB  ARG A 278       7.889  60.214 -31.234  1.00 49.84           C  <-- err: 0.0362
ATOM   2115  O   ARG A 278       9.338  58.604 -33.327  1.00 49.84           O  <-- err: 0.101
ATOM   2116  CG  ARG A 278       6.762  61.180 -31.556  1.00 49.84           C  <-- err: 0.0994
ATOM   2117  CD  ARG A 278       7.245  62.629 -31.073  1.00 49.84           C  <-- err: 0.0494
ATOM   2118  NE  ARG A 278       6.118  63.434 -31.073  1.00 49.84           N  <-- err: 0.1386 ***
ATOM   2119  NH1 ARG A 278       6.440  64.400 -28.819  1.00 49.84           N  <-- err: 0.1019
ATOM   2120  NH2 ARG A 278       4.669  65.044 -30.107  1.00 49.84           N  <-- err: 0.0996
ATOM   2121  CZ  ARG A 278       5.635  64.239 -29.946  1.00 49.84           C  <-- err: 0.0711
ATOM   2122  OXT ARG A 278       7.084  58.765 -33.971  1.00 49.84           O  <-- err: 0.0921
```

```
*************** AF-E9Q368-F1-model_v4.pdb max_error: 0.1386
***** original
ATOM   7901  N   LYS A1021       6.208 -28.582  19.381  1.00 89.32           N
ATOM   7902  CA  LYS A1021       7.291 -29.199  18.602  1.00 89.32           C
ATOM   7903  C   LYS A1021       6.780 -29.837  17.313  1.00 89.32           C
ATOM   7904  CB  LYS A1021       8.029 -30.203  19.497  1.00 89.32           C
ATOM   7905  O   LYS A1021       7.392 -29.641  16.258  1.00 89.32           O
ATOM   7906  CG  LYS A1021       9.283 -30.776  18.826  1.00 89.32           C
ATOM   7907  CD  LYS A1021       9.996 -31.736  19.783  1.00 89.32           C
ATOM   7908  CE  LYS A1021      11.220 -32.351  19.101  1.00 89.32           C
ATOM   7909  NZ  LYS A1021      11.959 -33.237  20.032  1.00 89.32           N
ATOM   7910  N   ASP A1022       5.657 -30.545  17.382  1.00 91.07           N
ATOM   7911  CA  ASP A1022       5.066 -31.238  16.237  1.00 91.07           C
ATOM   7912  C   ASP A1022       4.472 -30.284  15.186  1.00 91.07           C
ATOM   7913  CB  ASP A1022       4.025 -32.251  16.729  1.00 91.07           C
ATOM   7914  O   ASP A1022       4.388 -30.672  14.018  1.00 91.07           O
ATOM   7915  CG  ASP A1022       4.672 -33.417  17.482  1.00 91.07           C
ATOM   7916  OD1 ASP A1022       5.393 -34.212  16.824  1.00 91.07           O
ATOM   7917  OD2 ASP A1022       4.467 -33.497  18.708  1.00 91.07           O
ATOM   7918  N   TYR A1023       4.138 -29.043  15.569  1.00 94.10           N
ATOM   7919  CA  TYR A1023       3.638 -27.971  14.687  1.00 94.10           C
ATOM   7920  C   TYR A1023       4.602 -26.782  14.565  1.00 94.10           C
ATOM   7921  CB  TYR A1023       2.245 -27.522  15.142  1.00 94.10           C
ATOM   7922  O   TYR A1023       4.203 -25.664  14.229  1.00 94.10           O
ATOM   7923  CG  TYR A1023       1.229 -28.641  15.175  1.00 94.10           C
ATOM   7924  CD1 TYR A1023       0.688 -29.157  13.981  1.00 94.10           C
ATOM   7925  CD2 TYR A1023       0.837 -29.175  16.412  1.00 94.10           C
ATOM   7926  CE1 TYR A1023      -0.253 -30.205  14.032  1.00 94.10           C
ATOM   7927  CE2 TYR A1023      -0.077 -30.236  16.466  1.00 94.10           C
ATOM   7928  OH  TYR A1023      -1.562 -31.733  15.350  1.00 94.10           O
ATOM   7929  CZ  TYR A1023      -0.641 -30.741  15.279  1.00 94.10           C
***** protestar_80_140
ATOM   7901  N   LYS A1021       6.164 -28.612  19.412  1.00 89.32           N  <-- err: 0.0616
ATOM   7902  CA  LYS A1021       7.268 -29.164  18.584  1.00 89.32           C  <-- err: 0.0456
ATOM   7903  C   LYS A1021       6.808 -29.808  17.296  1.00 89.32           C  <-- err: 0.0437
ATOM   7904  CB  LYS A1021       8.050 -30.268  19.481  1.00 89.32           C  <-- err: 0.0702
ATOM   7905  O   LYS A1021       7.406 -29.624  16.261  1.00 89.32           O  <-- err: 0.0222
ATOM   7906  CG  LYS A1021       9.338 -30.751  18.837  1.00 89.32           C  <-- err: 0.0614
ATOM   7907  CD  LYS A1021       9.982 -31.717  19.803  1.00 89.32           C  <-- err: 0.0309
ATOM   7908  CE  LYS A1021      11.270 -32.361  19.159  1.00 89.32           C  <-- err: 0.0772
ATOM   7909  NZ  LYS A1021      11.914 -33.166  19.964  1.00 89.32           N  <-- err: 0.1081
ATOM   7910  N   ASP A1022       5.612 -30.544  17.388  1.00 91.07           N  <-- err: 0.0454
ATOM   7911  CA  ASP A1022       5.060 -31.280  16.192  1.00 91.07           C  <-- err: 0.0618
ATOM   7912  C   ASP A1022       4.508 -30.268  15.180  1.00 91.07           C  <-- err: 0.0398
ATOM   7913  CB  ASP A1022       4.025 -32.200  16.744  1.00 91.07           C  <-- err: 0.0532
ATOM   7914  O   ASP A1022       4.347 -30.751  14.007  1.00 91.07           O  <-- err: 0.0897
ATOM   7915  CG  ASP A1022       4.669 -33.488  17.549  1.00 91.07           C  <-- err: 0.0977
ATOM   7916  OD1 ASP A1022       5.313 -34.132  16.744  1.00 91.07           O  <-- err: 0.1386 ***
ATOM   7917  OD2 ASP A1022       4.508 -33.488  18.676  1.00 91.07           O  <-- err: 0.0528
ATOM   7918  N   TYR A1023       4.140 -29.072  15.548  1.00 94.10           N  <-- err: 0.0359
ATOM   7919  CA  TYR A1023       3.680 -27.968  14.720  1.00 94.10           C  <-- err: 0.0535
ATOM   7920  C   TYR A1023       4.600 -26.772  14.536  1.00 94.10           C  <-- err: 0.0307
ATOM   7921  CB  TYR A1023       2.254 -27.531  15.134  1.00 94.10           C  <-- err: 0.015
ATOM   7922  O   TYR A1023       4.186 -25.599  14.168  1.00 94.10           O  <-- err: 0.0907
ATOM   7923  CG  TYR A1023       1.288 -28.658  15.134  1.00 94.10           C  <-- err: 0.0738
ATOM   7924  CD1 TYR A1023       0.644 -29.141  14.007  1.00 94.10           C  <-- err: 0.0536
ATOM   7925  CD2 TYR A1023       0.805 -29.141  16.422  1.00 94.10           C  <-- err: 0.0477
ATOM   7926  CE1 TYR A1023      -0.322 -30.268  14.007  1.00 94.10           C  <-- err: 0.0967
ATOM   7927  CE2 TYR A1023       0.000 -30.268  16.422  1.00 94.10           C  <-- err: 0.0943
ATOM   7928  OH  TYR A1023      -1.610 -31.717  15.295  1.00 94.10           O  <-- err: 0.0747
ATOM   7929  CZ  TYR A1023      -0.644 -30.751  15.295  1.00 94.10           C  <-- err: 0.0191
```

5.6 ProteStAr 200/300

```
*************** AF-P70451-F1-model_v4.pdb max_error: 0.2991
***** original
ATOM   1141  N   ARG A 141      48.559  17.988 -64.900  1.00 96.64           N
ATOM   1142  CA  ARG A 141      48.524  19.428 -65.213  1.00 96.64           C
ATOM   1143  C   ARG A 141      49.918  20.051 -65.179  1.00 96.64           C
ATOM   1144  CB  ARG A 141      47.606  20.158 -64.225  1.00 96.64           C
ATOM   1145  O   ARG A 141      50.207  20.917 -66.004  1.00 96.64           O
ATOM   1146  CG  ARG A 141      46.121  19.830 -64.432  1.00 96.64           C
ATOM   1147  CD  ARG A 141      45.303  20.497 -63.320  1.00 96.64           C
ATOM   1148  NE  ARG A 141      43.898  20.054 -63.324  1.00 96.64           N
ATOM   1149  NH1 ARG A 141      43.238  21.178 -61.429  1.00 96.64           N
ATOM   1150  NH2 ARG A 141      41.802  19.826 -62.455  1.00 96.64           N
ATOM   1151  CZ  ARG A 141      42.990  20.355 -62.410  1.00 96.64           C
ATOM   1152  N   GLN A 142      50.776  19.615 -64.258  1.00 95.38           N
ATOM   1153  CA  GLN A 142      52.164  20.064 -64.200  1.00 95.38           C
ATOM   1154  C   GLN A 142      52.953  19.618 -65.440  1.00 95.38           C
ATOM   1155  CB  GLN A 142      52.806  19.580 -62.895  1.00 95.38           C
ATOM   1156  O   GLN A 142      53.563  20.459 -66.099  1.00 95.38           O
ATOM   1157  CG  GLN A 142      54.239  20.111 -62.771  1.00 95.38           C
ATOM   1158  CD  GLN A 142      54.934  19.686 -61.487  1.00 95.38           C
ATOM   1159  NE2 GLN A 142      56.226  19.895 -61.415  1.00 95.38           N
ATOM   1160  OE1 GLN A 142      54.342  19.165 -60.553  1.00 95.38           O
ATOM   1161  N   LEU A 143      52.884  18.336 -65.815  1.00 95.23           N
ATOM   1162  CA  LEU A 143      53.600  17.826 -66.988  1.00 95.23           C
ATOM   1163  C   LEU A 143      53.094  18.430 -68.305  1.00 95.23           C
ATOM   1164  CB  LEU A 143      53.537  16.292 -67.040  1.00 95.23           C
ATOM   1165  O   LEU A 143      53.898  18.638 -69.210  1.00 95.23           O
ATOM   1166  CG  LEU A 143      54.338  15.554 -65.952  1.00 95.23           C
ATOM   1167  CD1 LEU A 143      54.347  14.065 -66.290  1.00 95.23           C
ATOM   1168  CD2 LEU A 143      55.801  15.992 -65.861  1.00 95.23           C
***** protestar_200_300
ATOM   1141  N   ARG A 141      48.530  17.940 -64.860  1.00 96.64           N  <-- err: 0.0689
ATOM   1142  CA  ARG A 141      48.530  19.320 -65.320  1.00 96.64           C  <-- err: 0.1521
ATOM   1143  C   ARG A 141      49.910  20.010 -65.090  1.00 96.64           C  <-- err: 0.0983
ATOM   1144  CB  ARG A 141      47.748  20.068 -64.356  1.00 96.64           C  <-- err: 0.2131
ATOM   1145  O   ARG A 141      50.170  20.760 -66.086  1.00 96.64           O  <-- err: 0.1809
ATOM   1146  CG  ARG A 141      46.018  19.722 -64.356  1.00 96.64           C  <-- err: 0.1675
ATOM   1147  CD  ARG A 141      45.326  20.414 -63.318  1.00 96.64           C  <-- err: 0.0862
ATOM   1148  NE  ARG A 141      43.942  20.068 -63.318  1.00 96.64           N  <-- err: 0.0466
ATOM   1149  NH1 ARG A 141      43.250  21.106 -61.588  1.00 96.64           N  <-- err: 0.175
ATOM   1150  NH2 ARG A 141      41.866  19.722 -62.626  1.00 96.64           N  <-- err: 0.2101
ATOM   1151  CZ  ARG A 141      42.904  20.414 -62.280  1.00 96.64           C  <-- err: 0.1667
ATOM   1152  N   GLN A 142      50.830  19.550 -64.170  1.00 95.38           N  <-- err: 0.122
ATOM   1153  CA  GLN A 142      52.210  20.010 -64.170  1.00 95.38           C  <-- err: 0.077
ATOM   1154  C   GLN A 142      52.900  19.550 -65.550  1.00 95.38           C  <-- err: 0.1398
ATOM   1155  CB  GLN A 142      52.938  19.722 -62.972  1.00 95.38           C  <-- err: 0.2086
ATOM   1156  O   GLN A 142      53.630  20.414 -66.086  1.00 95.38           O  <-- err: 0.0817
ATOM   1157  CG  GLN A 142      54.322  20.068 -62.626  1.00 95.38           C  <-- err: 0.1725
ATOM   1158  CD  GLN A 142      55.014  19.722 -61.588  1.00 95.38           C  <-- err: 0.1338
ATOM   1159  NE2 GLN A 142      56.398  20.068 -61.588  1.00 95.38           N  <-- err: 0.2991 ***
ATOM   1160  OE1 GLN A 142      54.322  19.030 -60.550  1.00 95.38           O  <-- err: 0.1365
ATOM   1161  N   LEU A 143      52.900  18.400 -65.780  1.00 95.23           N  <-- err: 0.0747
ATOM   1162  CA  LEU A 143      53.590  17.940 -66.930  1.00 95.23           C  <-- err: 0.1283
ATOM   1163  C   LEU A 143      53.130  18.400 -68.310  1.00 95.23           C  <-- err: 0.0471
ATOM   1164  CB  LEU A 143      53.630  16.262 -67.124  1.00 95.23           C  <-- err: 0.1289
ATOM   1165  O   LEU A 143      53.976  18.684 -69.200  1.00 95.23           O  <-- err: 0.0911
ATOM   1166  CG  LEU A 143      54.322  15.570 -66.086  1.00 95.23           C  <-- err: 0.1359
ATOM   1167  CD1 LEU A 143      54.322  14.186 -66.432  1.00 95.23           C  <-- err: 0.1882
ATOM   1168  CD2 LEU A 143      55.706  15.916 -65.740  1.00 95.23           C  <-- err: 0.1716
```

```
*************** AF-Q8BW72-F1-model_v4.pdb max_error: 0.2991
***** original
ATOM   4632  N   MET A 584     -32.650  27.919 -17.563  1.00 57.33           N
ATOM   4633  CA  MET A 584     -31.968  28.384 -16.346  1.00 57.33           C
ATOM   4634  C   MET A 584     -32.774  29.456 -15.597  1.00 57.33           C
ATOM   4635  CB  MET A 584     -30.570  28.913 -16.705  1.00 57.33           C
ATOM   4636  O   MET A 584     -32.970  29.339 -14.387  1.00 57.33           O
ATOM   4637  CG  MET A 584     -29.549  27.784 -16.900  1.00 57.33           C
ATOM   4638  SD  MET A 584     -29.075  26.868 -15.396  1.00 57.33           S
ATOM   4639  CE  MET A 584     -28.293  28.204 -14.444  1.00 57.33           C
ATOM   4640  N   LEU A 585     -33.323  30.447 -16.300  1.00 56.08           N
ATOM   4641  CA  LEU A 585     -34.170  31.500 -15.738  1.00 56.08           C
ATOM   4642  C   LEU A 585     -35.447  30.915 -15.118  1.00 56.08           C
ATOM   4643  CB  LEU A 585     -34.476  32.548 -16.827  1.00 56.08           C
ATOM   4644  O   LEU A 585     -35.811  31.313 -14.014  1.00 56.08           O
ATOM   4645  CG  LEU A 585     -33.270  33.443 -17.186  1.00 56.08           C
ATOM   4646  CD1 LEU A 585     -33.545  34.211 -18.480  1.00 56.08           C
ATOM   4647  CD2 LEU A 585     -32.977  34.469 -16.086  1.00 56.08           C
ATOM   4648  N   SER A 586     -36.053  29.882 -15.717  1.00 46.98           N
ATOM   4649  CA  SER A 586     -37.198  29.175 -15.112  1.00 46.98           C
ATOM   4650  C   SER A 586     -36.871  28.498 -13.763  1.00 46.98           C
ATOM   4651  CB  SER A 586     -37.776  28.162 -16.109  1.00 46.98           C
ATOM   4652  O   SER A 586     -37.739  28.348 -12.893  1.00 46.98           O
ATOM   4653  OG  SER A 586     -37.075  26.929 -16.109  1.00 46.98           O
***** protestar_200_300
ATOM   4632  N   MET A 584     -32.660  27.830 -17.480  1.00 57.33           N  <-- err: 0.1221
ATOM   4633  CA  MET A 584     -31.970  28.290 -16.330  1.00 57.33           C  <-- err: 0.0954
ATOM   4634  C   MET A 584     -32.660  29.440 -15.640  1.00 57.33           C  <-- err: 0.1229
ATOM   4635  CB  MET A 584     -30.448  29.064 -16.608  1.00 57.33           C  <-- err: 0.217
ATOM   4636  O   MET A 584     -32.870  29.410 -14.532  1.00 57.33           O  <-- err: 0.1899
ATOM   4637  CG  MET A 584     -29.410  27.680 -16.954  1.00 57.33           C  <-- err: 0.1818
ATOM   4638  SD  MET A 584     -29.064  26.988 -15.224  1.00 57.33           S  <-- err: 0.21
ATOM   4639  CE  MET A 584     -28.372  28.372 -14.532  1.00 57.33           C  <-- err: 0.2054
ATOM   4640  N   LEU A 585     -33.350  30.360 -16.330  1.00 56.08           N  <-- err: 0.0959
ATOM   4641  CA  LEU A 585     -34.270  31.510 -15.640  1.00 56.08           C  <-- err: 0.1404
ATOM   4642  C   LEU A 585     -35.420  30.820 -15.180  1.00 56.08           C  <-- err: 0.1166
ATOM   4643  CB  LEU A 585     -34.600  32.524 -16.954  1.00 56.08           C  <-- err: 0.1791
ATOM   4644  O   LEU A 585     -35.984  31.486 -14.186  1.00 56.08           O  <-- err: 0.2991 ***
ATOM   4645  CG  LEU A 585     -33.216  33.562 -17.300  1.00 56.08           C  <-- err: 0.1734
ATOM   4646  CD1 LEU A 585     -33.562  34.254 -18.338  1.00 56.08           C  <-- err: 0.1493
ATOM   4647  CD2 LEU A 585     -32.870  34.600 -15.916  1.00 56.08           C  <-- err: 0.2398
ATOM   4648  N   SER A 586     -36.110  29.900 -15.640  1.00 46.98           N  <-- err: 0.0975
ATOM   4649  CA  SER A 586     -37.260  29.210 -15.180  1.00 46.98           C  <-- err: 0.0985
ATOM   4650  C   SER A 586     -36.800  28.520 -13.800  1.00 46.98           C  <-- err: 0.083
ATOM   4651  CB  SER A 586     -37.714  28.026 -16.262  1.00 46.98           C  <-- err: 0.2139
ATOM   4652  O   SER A 586     -37.714  28.372 -12.802  1.00 46.98           O  <-- err: 0.0974
ATOM   4653  OG  SER A 586     -37.022  26.988 -16.262  1.00 46.98           O  <-- err: 0.1723
```

```
*************** AF-Q8BMG7-F1-model_v4.pdb max_error: 0.2991
***** original
ATOM   7981  N   ILE A1016      20.843   8.698 -26.431  1.00 94.74           N
ATOM   7982  CA  ILE A1016      22.175   8.489 -27.017  1.00 94.74           C
ATOM   7983  C   ILE A1016      22.645   9.717 -27.807  1.00 94.74           C
ATOM   7984  CB  ILE A1016      22.177   7.212 -27.879  1.00 94.74           C
ATOM   7985  O   ILE A1016      23.827  10.049 -27.758  1.00 94.74           O
ATOM   7986  CG1 ILE A1016      21.965   5.981 -26.972  1.00 94.74           C
ATOM   7987  CG2 ILE A1016      23.493   7.066 -28.669  1.00 94.74           C
ATOM   7988  CD1 ILE A1016      21.596   4.733 -27.766  1.00 94.74           C
ATOM   7989  N   GLU A1017      21.755  10.432 -28.497  1.00 92.89           N
ATOM   7990  CA  GLU A1017      22.119  11.692 -29.156  1.00 92.89           C
ATOM   7991  C   GLU A1017      22.636  12.716 -28.132  1.00 92.89           C
ATOM   7992  CB  GLU A1017      20.934  12.283 -29.929  1.00 92.89           C
ATOM   7993  O   GLU A1017      23.679  13.325 -28.364  1.00 92.89           O
ATOM   7994  CG  GLU A1017      20.471  11.493 -31.163  1.00 92.89           C
ATOM   7995  CD  GLU A1017      19.180  12.077 -31.775  1.00 92.89           C
ATOM   7996  OE1 GLU A1017      18.659  11.499 -32.755  1.00 92.89           O
ATOM   7997  OE2 GLU A1017      18.633  13.068 -31.228  1.00 92.89           O
ATOM   7998  N   HIS A1018      21.979  12.853 -26.972  1.00 94.90           N
ATOM   7999  CA  HIS A1018      22.477  13.683 -25.869  1.00 94.90           C
ATOM   8000  C   HIS A1018      23.816  13.187 -25.317  1.00 94.90           C
ATOM   8001  CB  HIS A1018      21.441  13.757 -24.739  1.00 94.90           C
ATOM   8002  O   HIS A1018      24.710  14.000 -25.096  1.00 94.90           O
ATOM   8003  CG  HIS A1018      20.353  14.759 -24.996  1.00 94.90           C
ATOM   8004  CD2 HIS A1018      19.016  14.509 -25.132  1.00 94.90           C
ATOM   8005  ND1 HIS A1018      20.532  16.117 -25.120  1.00 94.90           N
ATOM   8006  CE1 HIS A1018      19.324  16.678 -25.309  1.00 94.90           C
ATOM   8007  NE2 HIS A1018      18.372  15.735 -25.318  1.00 94.90           N
***** protestar_200_300
ATOM   7981  N   ILE A1016      20.930   8.740 -26.450  1.00 94.74           N  <-- err: 0.0985
ATOM   7982  CA  ILE A1016      22.080   8.510 -26.910  1.00 94.74           C  <-- err: 0.1446
ATOM   7983  C   ILE A1016      22.540   9.660 -27.830  1.00 94.74           C  <-- err: 0.1217
ATOM   7984  CB  ILE A1016      22.144   7.266 -28.026  1.00 94.74           C  <-- err: 0.16
ATOM   7985  O   ILE A1016      23.874  10.034 -27.680  1.00 94.74           O  <-- err: 0.0923
ATOM   7986  CG1 ILE A1016      21.798   5.882 -26.988  1.00 94.74           C  <-- err: 0.1948
ATOM   7987  CG2 ILE A1016      23.528   6.920 -28.718  1.00 94.74           C  <-- err: 0.1579
ATOM   7988  CD1 ILE A1016      21.452   4.844 -27.680  1.00 94.74           C  <-- err: 0.2011
ATOM   7989  N   GLU A1017      21.850  10.350 -28.520  1.00 92.89           N  <-- err: 0.1276
ATOM   7990  CA  GLU A1017      22.080  11.730 -29.210  1.00 92.89           C  <-- err: 0.0767
ATOM   7991  C   GLU A1017      22.540  12.650 -28.060  1.00 92.89           C  <-- err: 0.137
ATOM   7992  CB  GLU A1017      21.106  12.456 -30.102  1.00 92.89           C  <-- err: 0.2991 ***
ATOM   7993  O   GLU A1017      23.528  13.494 -28.372  1.00 92.89           O  <-- err: 0.2268
ATOM   7994  CG  GLU A1017      20.414  11.418 -31.140  1.00 92.89           C  <-- err: 0.097
ATOM   7995  CD  GLU A1017      19.030  12.110 -31.832  1.00 92.89           C  <-- err: 0.1638
ATOM   7996  OE1 GLU A1017      18.684  11.418 -32.870  1.00 92.89           O  <-- err: 0.1429
ATOM   7997  OE2 GLU A1017      18.684  13.148 -31.140  1.00 92.89           O  <-- err: 0.1294
ATOM   7998  N   HIS A1018      22.080  12.880 -26.910  1.00 94.90           N  <-- err: 0.1215
ATOM   7999  CA  HIS A1018      22.540  13.570 -25.760  1.00 94.90           C  <-- err: 0.1692
ATOM   8000  C   HIS A1018      23.920  13.110 -25.300  1.00 94.90           C  <-- err: 0.1305
ATOM   8001  CB  HIS A1018      21.452  13.840 -24.912  1.00 94.90           C  <-- err: 0.1922
ATOM   8002  O   HIS A1018      24.566  13.840 -25.258  1.00 94.90           O  <-- err: 0.2694
ATOM   8003  CG  HIS A1018      20.414  14.878 -24.912  1.00 94.90           C  <-- err: 0.1579
ATOM   8004  CD2 HIS A1018      19.030  14.532 -25.258  1.00 94.90           C  <-- err: 0.1288
ATOM   8005  ND1 HIS A1018      20.414  16.262 -25.258  1.00 94.90           N  <-- err: 0.2324
ATOM   8006  CE1 HIS A1018      19.376  16.608 -25.258  1.00 94.90           C  <-- err: 0.101
ATOM   8007  NE2 HIS A1018      18.338  15.570 -25.258  1.00 94.90           N  <-- err: 0.1788
```

```
*************** AF-Q8CHC4-F1-model_v4.pdb max_error: 0.2991
***** original
ATOM   4919  N   LYS A 624      -0.907  18.960 -26.516  1.00 92.19           N
ATOM   4920  CA  LYS A 624      -0.538  19.120 -25.090  1.00 92.19           C
ATOM   4921  C   LYS A 624      -0.270  17.766 -24.408  1.00 92.19           C
ATOM   4922  CB  LYS A 624      -1.607  19.952 -24.348  1.00 92.19           C
ATOM   4923  O   LYS A 624      -1.088  16.849 -24.511  1.00 92.19           O
ATOM   4924  CG  LYS A 624      -1.275  20.187 -22.859  1.00 92.19           C
ATOM   4925  CD  LYS A 624      -2.231  21.202 -22.213  1.00 92.19           C
ATOM   4926  CE  LYS A 624      -2.012  21.295 -20.696  1.00 92.19           C
ATOM   4927  NZ  LYS A 624      -2.877  22.332 -20.086  1.00 92.19           N
ATOM   4928  N   TYR A 625       0.822  17.680 -23.642  1.00 94.11           N
ATOM   4929  CA  TYR A 625       1.097  16.600 -22.685  1.00 94.11           C
ATOM   4930  C   TYR A 625       1.323  17.165 -21.278  1.00 94.11           C
ATOM   4931  CB  TYR A 625       2.305  15.747 -23.109  1.00 94.11           C
ATOM   4932  O   TYR A 625       1.740  18.310 -21.102  1.00 94.11           O
ATOM   4933  CG  TYR A 625       2.155  15.047 -24.445  1.00 94.11           C
ATOM   4934  CD1 TYR A 625       1.732  13.705 -24.530  1.00 94.11           C
ATOM   4935  CD2 TYR A 625       2.431  15.765 -25.618  1.00 94.11           C
ATOM   4936  CE1 TYR A 625       1.535  13.108 -25.794  1.00 94.11           C
ATOM   4937  CE2 TYR A 625       2.230  15.181 -26.877  1.00 94.11           C
ATOM   4938  OH  TYR A 625       1.557  13.321 -28.198  1.00 94.11           O
ATOM   4939  CZ  TYR A 625       1.755  13.861 -26.969  1.00 94.11           C
ATOM   4940  N   VAL A 626       1.068  16.331 -20.273  1.00 94.15           N
ATOM   4941  CA  VAL A 626       1.326  16.615 -18.855  1.00 94.15           C
ATOM   4942  C   VAL A 626       2.168  15.501 -18.245  1.00 94.15           C
ATOM   4943  CB  VAL A 626       0.027  16.853 -18.061  1.00 94.15           C
ATOM   4944  O   VAL A 626       2.067  14.339 -18.655  1.00 94.15           O
ATOM   4945  CG1 VAL A 626      -0.696  18.103 -18.579  1.00 94.15           C
ATOM   4946  CG2 VAL A 626      -0.954  15.671 -18.111  1.00 94.15           C
***** protestar_200_300
ATOM   4919  N   LYS A 624      -0.920  18.860 -26.450  1.00 92.19           N  <-- err: 0.1205
ATOM   4920  CA  LYS A 624      -0.460  19.090 -25.070  1.00 92.19           C  <-- err: 0.0859
ATOM   4921  C   LYS A 624      -0.230  17.710 -24.380  1.00 92.19           C  <-- err: 0.0743
ATOM   4922  CB  LYS A 624      -1.730  20.068 -24.220  1.00 92.19           C  <-- err: 0.2121
ATOM   4923  O   LYS A 624      -1.038  16.954 -24.566  1.00 92.19           O  <-- err: 0.1286
ATOM   4924  CG  LYS A 624      -1.384  20.068 -22.836  1.00 92.19           C  <-- err: 0.163
ATOM   4925  CD  LYS A 624      -2.076  21.106 -22.144  1.00 92.19           C  <-- err: 0.1949
ATOM   4926  CE  LYS A 624      -2.076  21.452 -20.760  1.00 92.19           C  <-- err: 0.1812
ATOM   4927  NZ  LYS A 624      -2.768  22.490 -20.068  1.00 92.19           N  <-- err: 0.1928
ATOM   4928  N   TYR A 625       0.920  17.710 -23.690  1.00 94.11           N  <-- err: 0.1132
ATOM   4929  CA  TYR A 625       1.150  16.560 -22.770  1.00 94.11           C  <-- err: 0.1079
ATOM   4930  C   TYR A 625       1.380  17.250 -21.390  1.00 94.11           C  <-- err: 0.1517
ATOM   4931  CB  TYR A 625       2.422  15.916 -23.182  1.00 94.11           C  <-- err: 0.2181
ATOM   4932  O   TYR A 625       1.730  18.338 -21.106  1.00 94.11           O  <-- err: 0.03
ATOM   4933  CG  TYR A 625       2.076  14.878 -24.566  1.00 94.11           C  <-- err: 0.2224
ATOM   4934  CD1 TYR A 625       1.730  13.840 -24.566  1.00 94.11           C  <-- err: 0.1397
ATOM   4935  CD2 TYR A 625       2.422  15.916 -25.604  1.00 94.11           C  <-- err: 0.1519
ATOM   4936  CE1 TYR A 625       1.384  13.148 -25.950  1.00 94.11           C  <-- err: 0.2208
ATOM   4937  CE2 TYR A 625       2.076  15.224 -26.988  1.00 94.11           C  <-- err: 0.1946
ATOM   4938  OH  TYR A 625       1.730  13.494 -28.026  1.00 94.11           O  <-- err: 0.2991 ***
ATOM   4939  CZ  TYR A 625       1.730  13.840 -26.988  1.00 94.11           C  <-- err: 0.0378
ATOM   4940  N   VAL A 626       1.150  16.330 -20.240  1.00 94.15           N  <-- err: 0.0884
ATOM   4941  CA  VAL A 626       1.380  16.560 -18.860  1.00 94.15           C  <-- err: 0.0772
ATOM   4942  C   VAL A 626       2.070  15.410 -18.170  1.00 94.15           C  <-- err: 0.1533
ATOM   4943  CB  VAL A 626       0.000  16.954 -17.992  1.00 94.15           C  <-- err: 0.1253
ATOM   4944  O   VAL A 626       2.076  14.186 -18.684  1.00 94.15           O  <-- err: 0.156
ATOM   4945  CG1 VAL A 626      -0.692  17.992 -18.684  1.00 94.15           C  <-- err: 0.1528
ATOM   4946  CG2 VAL A 626      -1.038  15.570 -17.992  1.00 94.15           C  <-- err: 0.1773
```

```
*************** AF-O35386-F1-model_v4.pdb max_error: 0.2991
***** original
ATOM    617  N   ARG A  80       6.255  16.845   6.733  1.00 98.75           N
ATOM    618  CA  ARG A  80       7.582  16.306   7.056  1.00 98.75           C
ATOM    619  C   ARG A  80       7.944  15.115   6.168  1.00 98.75           C
ATOM    620  CB  ARG A  80       7.656  15.917   8.531  1.00 98.75           C
ATOM    621  O   ARG A  80       9.023  15.113   5.582  1.00 98.75           O
ATOM    622  CG  ARG A  80       7.583  17.109   9.494  1.00 98.75           C
ATOM    623  CD  ARG A  80       7.449  16.632  10.944  1.00 98.75           C
ATOM    624  NE  ARG A  80       8.594  15.802  11.370  1.00 98.75           N
ATOM    625  NH1 ARG A  80       8.137  15.753  13.610  1.00 98.75           N
ATOM    626  NH2 ARG A  80      10.010  14.814  12.834  1.00 98.75           N
ATOM    627  CZ  ARG A  80       8.912  15.465  12.601  1.00 98.75           C
ATOM    628  N   PHE A  81       7.020  14.174   5.957  1.00 98.74           N
ATOM    629  CA  PHE A  81       7.241  13.050   5.037  1.00 98.74           C
ATOM    630  C   PHE A  81       7.461  13.512   3.587  1.00 98.74           C
ATOM    631  CB  PHE A  81       6.047  12.082   5.093  1.00 98.74           C
ATOM    632  O   PHE A  81       8.284  12.944   2.870  1.00 98.74           O
ATOM    633  CG  PHE A  81       5.678  11.515   6.453  1.00 98.74           C
ATOM    634  CD1 PHE A  81       6.658  11.264   7.433  1.00 98.74           C
ATOM    635  CD2 PHE A  81       4.326  11.245   6.747  1.00 98.74           C
ATOM    636  CE1 PHE A  81       6.278  10.786   8.696  1.00 98.74           C
ATOM    637  CE2 PHE A  81       3.953  10.757   8.012  1.00 98.74           C
ATOM    638  CZ  PHE A  81       4.933  10.530   8.991  1.00 98.74           C
ATOM    639  N   ARG A  82       6.745  14.554   3.131  1.00 98.26           N
ATOM    640  CA  ARG A  82       6.961  15.148   1.797  1.00 98.26           C
ATOM    641  C   ARG A  82       8.344  15.779   1.686  1.00 98.26           C
ATOM    642  CB  ARG A  82       5.902  16.211   1.458  1.00 98.26           C
ATOM    643  O   ARG A  82       9.019  15.546   0.687  1.00 98.26           O
ATOM    644  CG  ARG A  82       4.524  15.605   1.185  1.00 98.26           C
ATOM    645  CD  ARG A  82       3.462  16.667   0.893  1.00 98.26           C
ATOM    646  NE  ARG A  82       3.644  17.254  -0.447  1.00 98.26           N
ATOM    647  NH1 ARG A  82       1.947  16.144  -1.575  1.00 98.26           N
ATOM    648  NH2 ARG A  82       3.294  17.552  -2.666  1.00 98.26           N
ATOM    649  CZ  ARG A  82       2.958  16.980  -1.545  1.00 98.26           C
***** protestar_200_300
ATOM    617  N   ARG A  80       6.210  16.790   6.670  1.00 98.75           N  <-- err: 0.095
ATOM    618  CA  ARG A  80       7.590  16.330   7.130  1.00 98.75           C  <-- err: 0.0782
ATOM    619  C   ARG A  80       8.050  15.180   6.210  1.00 98.75           C  <-- err: 0.1312
ATOM    620  CB  ARG A  80       7.612  15.916   8.650  1.00 98.75           C  <-- err: 0.1269
ATOM    621  O   ARG A  80       8.996  15.224   5.536  1.00 98.75           O  <-- err: 0.1232
ATOM    622  CG  ARG A  80       7.612  16.954   9.342  1.00 98.75           C  <-- err: 0.219
ATOM    623  CD  ARG A  80       7.612  16.608  11.072  1.00 98.75           C  <-- err: 0.2086
ATOM    624  NE  ARG A  80       8.650  15.916  11.418  1.00 98.75           N  <-- err: 0.1358
ATOM    625  NH1 ARG A  80       8.304  15.916  13.494  1.00 98.75           N  <-- err: 0.2606
ATOM    626  NH2 ARG A  80      10.034  14.878  12.802  1.00 98.75           N  <-- err: 0.0755
ATOM    627  CZ  ARG A  80       8.996  15.570  12.456  1.00 98.75           C  <-- err: 0.1978
ATOM    628  N   PHE A  81       7.130  14.260   5.980  1.00 98.74           N  <-- err: 0.1415
ATOM    629  CA  PHE A  81       7.130  13.110   5.060  1.00 98.74           C  <-- err: 0.1283
ATOM    630  C   PHE A  81       7.360  13.570   3.680  1.00 98.74           C  <-- err: 0.149
ATOM    631  CB  PHE A  81       5.882  12.110   5.190  1.00 98.74           C  <-- err: 0.1934
ATOM    632  O   PHE A  81       8.304  12.802   2.768  1.00 98.74           O  <-- err: 0.176
ATOM    633  CG  PHE A  81       5.536  11.418   6.574  1.00 98.74           C  <-- err: 0.2103
ATOM    634  CD1 PHE A  81       6.574  11.418   7.266  1.00 98.74           C  <-- err: 0.2422
ATOM    635  CD2 PHE A  81       4.498  11.418   6.920  1.00 98.74           C  <-- err: 0.2991 ***
ATOM    636  CE1 PHE A  81       6.228  10.726   8.650  1.00 98.74           C  <-- err: 0.0906
ATOM    637  CE2 PHE A  81       3.806  10.726   7.958  1.00 98.74           C  <-- err: 0.1596
ATOM    638  CZ  PHE A  81       4.844  10.380   8.996  1.00 98.74           C  <-- err: 0.1745
ATOM    639  N   ARG A  82       6.670  14.490   3.220  1.00 98.26           N  <-- err: 0.1328
ATOM    640  CA  ARG A  82       6.900  15.180   1.840  1.00 98.26           C  <-- err: 0.0812
ATOM    641  C   ARG A  82       8.280  15.870   1.610  1.00 98.26           C  <-- err: 0.1347
ATOM    642  CB  ARG A  82       5.882  16.262   1.384  1.00 98.26           C  <-- err: 0.0921
ATOM    643  O   ARG A  82       8.996  15.570   0.692  1.00 98.26           O  <-- err: 0.0336
ATOM    644  CG  ARG A  82       4.498  15.570   1.038  1.00 98.26           C  <-- err: 0.1533
ATOM    645  CD  ARG A  82       3.460  16.608   1.038  1.00 98.26           C  <-- err: 0.1566
ATOM    646  NE  ARG A  82       3.806  17.300  -0.346  1.00 98.26           N  <-- err: 0.1964
ATOM    647  NH1 ARG A  82       2.076  16.262  -1.730  1.00 98.26           N  <-- err: 0.2336
ATOM    648  NH2 ARG A  82       3.460  17.646  -2.768  1.00 98.26           N  <-- err: 0.2163
ATOM    649  CZ  ARG A  82       3.114  16.954  -1.384  1.00 98.26           C  <-- err: 0.2257
```
